# A cell-based chemical-genetic screen for amino acid stress response inhibitors reveals torins reverse stress kinase GCN2 signaling

**DOI:** 10.1016/j.jbc.2022.102629

**Published:** 2022-10-20

**Authors:** Johanna B. Brüggenthies, Alessandra Fiore, Marion Russier, Christina Bitsina, Julian Brötzmann, Susanne Kordes, Sascha Menninger, Alexander Wolf, Elena Conti, Jan E. Eickhoff, Peter J. Murray

**Affiliations:** 1 Max Planck Institute for Biochemistry, Martinsried, Germany; 2 Lead Discovery Center GmbH, Dortmund, Germany

**Keywords:** GCN2, mTORC1, torins, amino acid starvation, integrated stress response, BSA, bovine serum albumin, CHX, cycloheximide, DMSO, dimethyl sulfoxide, ER, endoplasmic reticulum, IP, immunoprecipitation, ISR, integrated stress response, Tg, thapsigargin

## Abstract

mTORC1 and GCN2 are serine/threonine kinases that control how cells adapt to amino acid availability. mTORC1 responds to amino acids to promote translation and cell growth while GCN2 senses limiting amino acids to hinder translation *via* eIF2α phosphorylation. GCN2 is an appealing target for cancer therapies because malignant cells can harness the GCN2 pathway to temper the rate of translation during rapid amino acid consumption. To isolate new GCN2 inhibitors, we created cell-based, amino acid limitation reporters *via* genetic manipulation of *Ddit3* (encoding the transcription factor CHOP). CHOP is strongly induced by limiting amino acids and in this context, GCN2-dependent. Using leucine starvation as a model for essential amino acid sensing, we unexpectedly discovered ATP-competitive PI3 kinase-related kinase inhibitors, including ATR and mTOR inhibitors like torins, completely reversed GCN2 activation in a time-dependent way. Mechanistically, *via* inhibiting mTORC1-dependent translation, torins increased intracellular leucine, which was sufficient to reverse GCN2 activation and the downstream integrated stress response including stress-induced transcriptional factor ATF4 expression. Strikingly, we found that general translation inhibitors mirrored the effects of torins. Therefore, we propose that mTOR kinase inhibitors concurrently inhibit different branches of amino acid sensing by a dual mechanism involving direct inhibition of mTOR and indirect suppression of GCN2 that are connected by effects on the translation machinery. Collectively, our results highlight distinct ways of regulating GCN2 activity.

Sufficient amino acid amounts sustain protein homeostasis and interconnected metabolic pathways that control virtually all aspects of cellular physiology (1, 2). Eukaryotic cells detect amino acid amounts inside and outside cells and then integrate this information to control the balance between growth *versus* cellular stress adaptation pathways (3, 4). A major amino acid sensing and signaling pathway is regulated by mTORC1 (5), an essential progrowth and proliferative multiprotein signaling hub that is highly sensitive to amino acid availability and especially leucine (Leu) and arginine (Arg) (6). When key amino acids are limiting, amino acid–sensing proteins transiently repress the activity of mTORC1 (6, 7). For example, at least four proteins act as Leu sensors (LARS, sestrin-2, SAR1B, and TSC) to transfer information about available Leu amounts by direct Leu binding (LARS, sestrin-2, and SAR1B) or by indirect pathways (6, 7, 8, 9, 10, 11, 12). By sensing sufficient nutrients including amino acids, mTORC1 positively regulates translation by pathways that include stimulating the activity of the p70 S6 kinase, which phosphorylates the ribosome S6 subunit, and by promoting cap-dependent translation *via* 4EBP1 phosphorylation at threonine 37/46 (T37/46, in mouse T36/45) (13, 14). The connection between nutrient sensing and mTORC1 activity occurs in a temporal and transient way: upon short-term amino acid restriction (minutes), mTORC1 is suppressed. Later (hours), mTORC1 activity (in terms of kinase activity toward S6K and 4EBP1) returns, in part *via* mechanisms that supply free amino acids harvested from different cellular and extracellular sources linked to glutamine metabolism, energy sensing, and autophagy (15, 16, 17, 18, 19, 20, 21, 22). The cellular and biochemical nature of how the different amino acid sensors ultimately control mTORC1 remains largely incomplete, as does the dynamics and cellular activity of mTORC1 under prolonged nutrient starvation (6, 23).

Signaling through the mTOR pathway is essential for proliferation of all cells and *Mtor* is a core essential gene. Aberrant or sustained mTOR signaling through the mTORC1 and mTORC2 complexes license anabolic growth and proliferation and thereby contribute to malignancy. Ergo, development of mTOR inhibitors could have important anticancer effects. Pharmacological suppression of mTORC1 includes two types of inhibitors. First, the macrolide rapamycin and its analogs such as everolimus are ‘rapalogs’, which are the first generation of allosteric mTORC1 inhibitors that bind to the FKBP12 component of the mTORC1 complex and modify substrate recruitment to the mTOR kinase domain (24). Second, mTOR kinase inhibitors or ‘TORKinibs’, are ATP-competitive inhibitors of both mTOR complexes (mTORC1 and mTORC2) and include torin-1, torin-2, and sapanisertib (also known as INK-128, MLN0128, TAK-228) (25, 26, 27). A bivalent hybrid inhibitor, RapaLink-1 comprises rapamycin linked to sapanisertib (28, 29, 30). Hereafter, mTOR ATP-competitive inhibitors (and their related compounds) are generically termed ‘torins’ for simplicity.

Another amino acid–sensing pathway is activated by the GCN2 kinase (General Control Non-derepressible 2, hereafter GCN2, encoded by *Eif2ak4*) (4, 6). When amino acid amounts become restricted, GCN2 is activated by an incompletely understood pathway that involves increased amounts of deacylated tRNAs that may directly bind to the histidyl-tRNA synthase like domain of GCN2, the ribosomal P-stalk, and the HEAT repeat protein GCN1, which converge to stimulate the dimerization and *trans*-autophosphorylation of GCN2 at threonine T899 (p-GCN2, T899, in mouse T898) in its kinase activation loop (3, 31, 32, 33, 34, 35, 36, 37). GCN2 is one of four mammalian integrated stress response (ISR) kinases (HRI, PERK, PKR, and GCN2), which respond to specific stress stimuli and repress cap-dependent translation initiation by phosphorylating their common target, the eukaryotic translation initiation factor eIF2α on serine 51 (p-eIF2α, S51, in mouse S52), a component of the ‘ternary complex’ that loads tRNA^MET^ into the ribosome to initiate translation (31, 34, 38, 39, 40). Concurrent with suppression of translation *via* eIF2α phosphorylation, the ISR kinases promote the expression of stress protective proteins including the B-ZIP transcription factor ATF4, which control the expression of hundreds-thousands of other ISR genes including the proapoptotic gene *Ddit3* and genes necessary for stress protection and resource conservation (41). The precise roles of the ISR response genes and how their protein products work in networks of stress protection are incomplete. Thus, when amino acids are limited, the effect of reduced mTORC1 activity on 4EBP1, combined with parallel GCN2-mediated phosphorylation of eIF2α partly represses translation (4).

Like mTORC1, GCN2 is also an appealing target for cancer drugs, which is based on the rationale that in contrast to mTOR, GCN2 is nonessential in normal cells and animals but that malignant cells have exaggerated amino acid dependencies and therefore use the GCN2-ISR as an adaptive survival and metabolic defense mechanism (42, 43). Perturbing the translational quality control by manipulating the GCN2-ISR has emerged as a therapeutic strategy in combination treatment with asparaginase (ASNase), an enzyme that depletes asparagine and glutamine, in acute lymphoblastic leukemia, highlighting the potential for GCN2 inhibitors (GCN2i) such as GCN2-IN-1, GCN2-IN-6, and GCN2iB in amino acid dependencies in cancer (44, 45). However, each of these inhibitors has shortcomings of specificity and potency (discussed later). Therefore, we designed a cell-based approach to identify new GCN2i. The outcome of this screen uncovered an unexpected intersection between the GCN2 and the mTORC1 branches of amino acid signaling.

## Results

### Amino acid–dependent switch-like behavior of the GCN2 ISR

**Figure 1:**
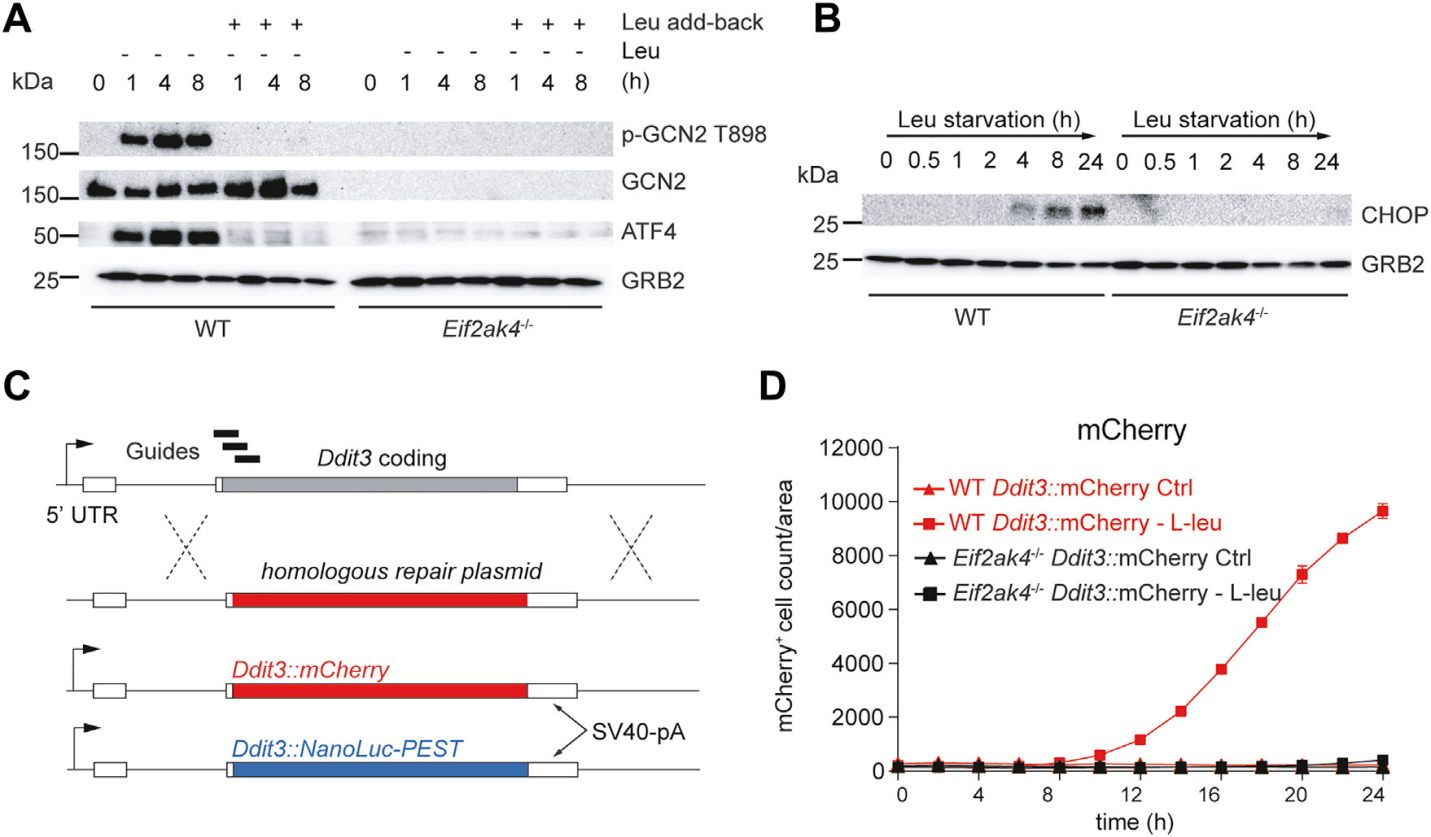
**GCN2 amino acid stress signaling genetic reporters.***A*, pathway immunoblotting analysis of the mammalian GCN2-ISR. 3T3 WT and GCN2-deficient (*Eif2ak4*^−/−^) cells were starved in Leu-deficient media (Leu starvation) for the times indicated (h). Lysates were probed for activation of GCN2 (p-GCN2 T898), total GCN2, ATF4 as a marker of the ISR. GRB2 was used as a loading control. *B*, kinetics and GCN2 dependency of CHOP expression in response to leucine starvation. Data are representative of a minimum of three independent experiments. *C*, schematic representation of the *Ddit3* locus (encoding CHOP) with the position of the sgRNAs (guides). Homologous repair plasmids are indicated. *D*, representative live-cell imaging showing the mCherry signal following Leu starvation and the dependence on GCN2. sgRNA, single-guide RNA.

We used NIH-3T3 (3T3) murine fibroblasts as genetically tractable model to monitor the dynamics of GCN2 pathway activation upon amino acid deprivation. As expected, leucine (an essential amino acid, hereafter Leu) starvation triggered a time-dependent activation of the GCN2 cascade, starting from GCN2 autophosphorylation at T898, followed by ATF4 and was reversible by the addition of leucine (Fig. 1*A*). CHOP (C/EBP Homologous Protein, encoded by *Ddit3*) expression, which is dependent on ATF4, was induced after ATF4. As expected, ATF4 and CHOP expression following leucine starvation was dependent on GCN2 (Fig. 1*B*).

### *Ddit3* reports ISR activation

CHOP is a downstream ATF4-dependent target of GCN2 (31, 46). We chose CHOP as an amino acid starvation reporter platform based on the principle that CHOP expression at the mRNA and protein level is GCN2-dependent and temporally predictable upon amino acid limitation (Fig. 1*B*). CHOP expression bypasses translational inhibition induced *via* p-eIF2α, which is mediated in part by the structure of the 5′ UTR of the CHOP mRNA (47, 48). CHOP expression is therefore independent of amino acid modulation of mTORC1 signaling (49). Using Crispr/Cas9-mediated mutagenesis and homologous recombination repair, we inserted reporters for fluorescence (mCherry) or chemiluminescence (NanoLuc-PEST) into the *Ddit3* coding locus by introducing mutations into the 5′ end of one allele of exon 2 of *Ddit3* (Figs. 1*C* and S1*A*). We tracked mCherry expression by live-cell imaging in WT and GCN2 (*Eif2ak4*^-/-^)-deficient *Ddit3*::mCherry reporter cells for 24 h of Leu starvation, which showed a temporal, two orders of magnitude increase in mCherry expression, dependent on GCN2 and Leu or Arg starvation stress (Figs. 1*D*, S1, *B*, and *C*). Similarly, the NanoLuc-PEST reporter was induced ∼8-fold above background, which was a sufficient dynamic range to screen for GCN2i (Fig. S1*D*).

The four ISR kinases share conserved kinase domains (Fig. S1*E*) and, once activated, all regulate CHOP expression. Ergo, a kinase inhibitor that targeted GCN2 could promiscuously inhibit other ISR kinases and especially PERK, which responds to the accumulation of misfolded and unfolded endoplasmic reticulum (ER) proteins and is the most likely ISR pathway that could be activated in parallel in amino acid restricted cells (4). We therefore determined if the activation of the PERK- and GCN2-ISR can be distinguished when either amino acid starvation (Leu and Arg stress) *versus* ER stress (thapsigargin (Tg) treatment; an inhibitor of the sarco/ER Ca^2+^ ATPase) was induced. *Ddit3*::mCherry expression was induced by Tg or by Leu starvation as expected (Fig. S1*F*). However, reporter expression was also induced by Tg in the GCN2-deficient *Ddit3*::mCherry background, while (as noted before) amino acid starvation-induced mCherry expression was completely ablated in the absence of GCN2. An anti-PERK phospho-specific antibody (T980) identified active PERK only when cells were treated with Tg, regardless of the presence or absence of GCN2 (Fig. S1*G*). In line, ATF4 and CHOP expression had predictable expression patterns that correlated to the cognate stress stimuli. These results underline the specificity and robustness of ISR pathway tracing in the 3T3 cell system at both the reporter and signaling pathway levels.

### GCN2 pathway inhibitors

To identify specific GCN2i, we performed a small molecule-based screen containing 3876 bioactive reference compounds including many kinase inhibitors (Table S1 and Fig. S2*A*), which were screened using the *Ddit3*::NanoLuc-PEST reporter cell system upon Leu starvation for 28 h. In this context, we identified 58 compounds with a IC_50_ lower than 30 μM, which were classified based on their structure and mode of action and further reduced to 28 hits for further evaluation (Table S1).

### GCN2 and mTORC1 signaling pathway evaluation

Concurrent with reporter-based small molecule screening, we incorporated GCN2 and mTORC1 pathway analysis for the best screen hits to discern which points(s) of the GCN2 pathway were inhibited. To simplify our approach but at the same time provide a sufficiently robust readout of the key steps, we focused on criteria that (i) reported GCN2 activation (p-GCN2 T899), (ii) ISR activation downstream of GCN2 (ATF4 expression), and (iii) mTORC1 activity (p4EBP1 T36/45). In part, these three targets were chosen because of the specificity and robustness of the antibodies we used. Using a starting inhibitor concentration of 10 μM, we identified compounds causing loss of p-GCN2 T899 phosphorylation, which matched to a reduction in ATF4 (*e*.*g*., compounds #12, #18, and #23) (Fig. S2*B*). Probing for total GCN2 amounts indicated that the loss of p-GCN2 T899 was not due to activation of a pathway(s) that could have degraded GCN2, such as *via* an E3 ligase. We noted that several compounds increased p-GCN2 T899 amounts relative to the controls (*e*.*g*., compounds #4, #6, and #7). This finding is consistent with a recent study that showed some kinase inhibitors activate GCN2 activity (50). Importantly, several compounds that showed increased p-GCN2 T899 amounts had reduced ATF4 (especially #6), accounting for their detection in the screen since CHOP expression is ATF4 dependent. As we could not readily account for compounds that elicited the activation of GCN2 while suppressing amino acid–induced ATF4 expression, we focused on those compounds that showed clear inhibition of p-GCN2 T899 and ATF4 expression.

**Figure 2:**
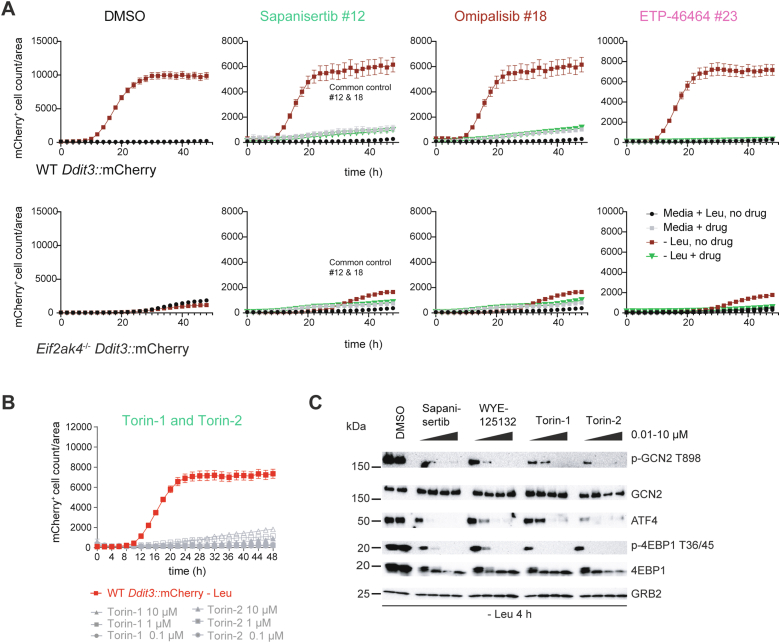
**Torins inhibit the GCN2 pathway.***A*, effect of sapanisertib, omipalisib, or ETP-46464 on *Ddit3*::mCherry activity. Drugs were used at 1 μM. Shown in each panel are WT Ddti3::mCherry 3T3 and GCN2-deficient cells (*Eif2ak4*^-/-^) untreated (Ctrl) over time and normalized to cellular confluence. The first panel (DMSO control) indicated the control for effects of the solvent. Note that a common control was used for sapanisertib and omipalisib. *B*, torin-1 or torin-2 was added to *Ddti3*::mCherry 3T3 cells in Leu-free media at 0.1, 1, or 10 μM and mCherry signal recorded and normalized to confluence. *C*, pathway immunoblotting analysis of the effect of torins on the GCN2 ISR *versus* the mTORC1 pathway. 3T3 cells were starved in Leu-deficient media for 4 h in the presence of the drugs indicated (0.01–10 μM) added at time zero. Lysates were probed for activation of GCN2 (p-GCN2 T898), total GCN2, ATF4, p-4EBP1 T36/45, or total 4EBP1. GRB2 was used as a loading control. Data are representative of at least five independent experiments. DMSO, dimethyl sulfoxide.

The majority of compounds that inhibited p-GCN2 T899 and ATF4 expression were ATP-competitive inhibitors of the PI3K and the class-IV PI3K-related kinase (PIKK) family that comprises mTOR, ATM, PRKDC, ATR, TRRAP, and SMG1 (51), which are unrelated to the ISR kinases both in structure and substrate specificity (25, 28). We found that the mTOR inhibitor (mTORi) sapanisertib (compound #12), the PI3K inhibitor (PI3Ki) omipalisib (compound #18) and the ATR inhibitor (ATRi) ETP-46464 (compound #23) almost completely inhibited CHOP reporter expression in a dose-dependent way (52) (Fig. 2*A*), as did torin-1 and torin-2 (Fig. 2*B*). Importantly, sapanisertib, WYE-125132, torin-1, and torin-2 also inhibited GCN2 activation (p-GCN2 T899), ISR activation downstream of GCN2 (ATF4 expression), and mTORC1 activity (p4EBP1 T36/45) (Fig. 2*C*), suggesting these torin class PIKK inhibitors have a dual activity of simultaneously inhibiting the mTORC1 and GCN2 when amino acid stress signaling is active. As additional controls, we compared three GCN2i (GCN2-IN-1, GCN2-IN-6, and GCN2iB) for their effects on the GCN2 ISR and mTORC1 (Fig. S2*D*). GCN2i inhibited p-GCN2 T899 and AFT4 expression with different potencies. Importantly, the two strongest GCN2i (GCN2-IN-6 and GCN2iB) did not inhibit p-4EBP1 T36/45. Another compound we isolated in the screen, (#25, GZD824; synonyms: olverembatinib, HQP1351) was recently described to be a GCN2i (53). In our hands, however, GZD824 was rapidly toxic to murine fibroblasts, independent on amino acid stress (Fig. S2, *E* and *F*). Finally, the results of dual GCN2 and mTORC1 pathway inhibition in 3T3 cells were confirmed in HeLa cells (human) and murine ES cells (Fig. S3, *A*–*C*).

### ATR inhibitors differentially block GCN2

Inhibition of PIKK family member ATR is a promising cancer treatment strategy for tumors that manifest ATR-dependent DNA damage response defects and replication stress (54, 55, 56). In our screen, the ATRi ETP-46464 (compound #23) was detected and suppressed p-GCN2 T899 and ATF4 expression upon amino acid stress (Fig. S2, *B* and *C*). We therefore compared ETP-46464 with other ATR inhibitors including BAY1895344 and berzosertib in the context of amino acid stress-induced GCN2-ISR perturbation. BAY1895344 or berzosertib inhibited *Ddit3*::mCherry activity, p-GCN2 and ATF4 expression at > 1 μM (Fig. S4*A*). At the same time, BAY1895344 or ETP-46464 also blocked mTORC1 activity (p-4EBP1 T36/45) in 3T3 or HeLa cells (Fig. S4, *B* and *C*). By contrast, berzosertib did not inhibit either the GCN2 and the mTOR pathway in either 3T3 or HeLa cells, suggesting a possible correlation between the specificity of the PIKK inhibitor: a highly specific ATRi like berzosertib had minimal activity toward either the mTORC1 or GCN2 pathways, while ETP-46464 inhibited both pathways.

### mTOR inhibitors are not direct GCN2 inhibitors

**Figure 3:**
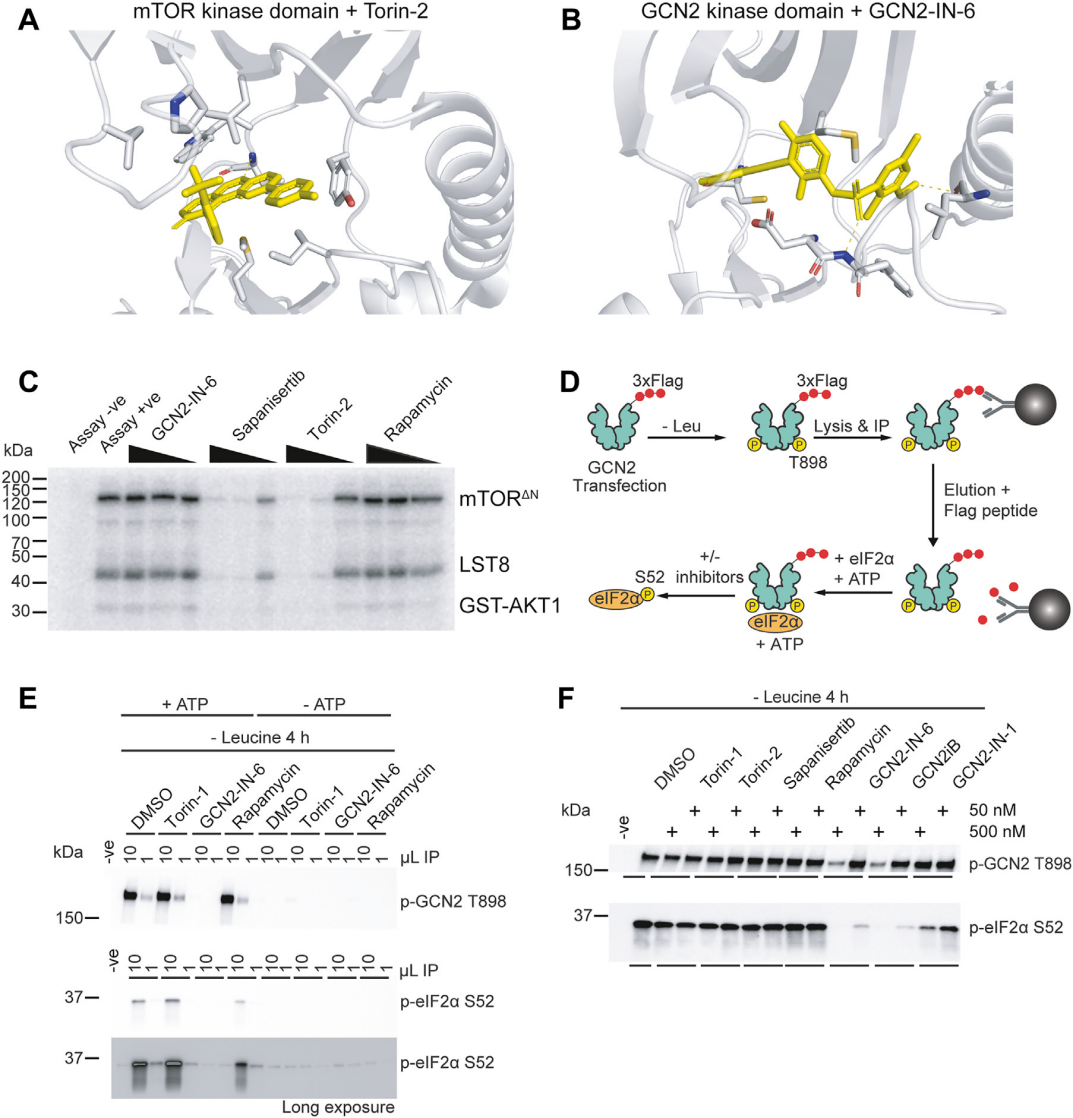
**Effect of torins on GCN2 activity.***A* and *B*, structure of mTOR kinase domain bound to torin-2 and the GCN2 kinase domain bound to GCN2-IN-6. Data were modeled in PyMol from PDB data 4JSX and 6N3N, respectively. *C*, *in vitro* mTOR kinase assay. Recombinant mTOR kinase domain in complex with full-length mLST8 was incubated with a model AKT substrate GST fusion in the presence of inhibitors (rapamycin, GCN2-IN-6, sapanisertib, torin-2, or rapamycin) titrated to 200, 100, or 10 nM. Data are representative of one of three independent experiments. *D*, schematic design of the *in vitro* GCN2 IP-kinase assay. *E*, GCN2 IP-kinase assay is dependent on input material (1 or 10 μl of IP material) and ATP. p-GCN2 T898 or p-eIF2α S52 (human recombinant protein) was analyzed by immunoblotting. *F*, *in vitro* GCN2 IP-kinase assay where active GCN2 was isolated from Leu starved cells for 4 h and assayed for inhibitor sensitivity analysis. Inhibitors were added at 0.05 μM or 0.5 μM for the assay period. GCN2 kinase data are representative of six independent experiments performed to quantify the effect of torins on GCN2 activity. IP, immunoprecipitation; PDB, Protein Data Bank.

We next tested the possibility that torins directly inhibited both mTOR and GCN2 enzyme activity. *A priori*, this possibility seemed unlikely as PIKKs and ISR kinases are distinct families with distinct substrate specifies. Furthermore, structural studies of bound torin-2 to mTOR and GCN2-IN-6 to the human GCN2 kinase domain show both distinctive enzymatic cleft and inhibitor architecture (Fig. 3, *A* and *B*). Nevertheless, we experimental tested if the GCN2i GCN2-IN-6 inhibited mTOR or if torins inhibited GCN2, by using kinase specificity assays. We first used an N-terminal–truncated active version of mTOR (mTOR^ΔN^) in complex with mLST8 (51), which is a promiscuous kinase *in vitro* (*i.e*., mTOR^ΔN^-mLST8 phosphorylates itself as well as introduced substrate, in this case GST-AKT1). GCN2-IN-6 did not repress mTOR kinase activity by contrast with torin-2 or sapanisertib consistent with the fact that GCN2-IN-6 had no modulatory effect on the mTORC1 pathway (Fig. 3*C*). Rapamycin did not inhibit mTOR kinase activity because FKBP12 is not a component of the assay system (24) (Fig. 3*C*).

We next performed reciprocal experiments to test the inhibitory effect of torins on GCN2 kinase activity. Using an *in vitro* immunoprecipitation-kinase (IP-kinase) assay with active GCN2 isolated from the cellular environment (40, 57), we isolated tagged GCN2 from Leu-starved cells and exposed the immunoprecipitated material to recombinant human eIF2α followed by measuring eIF2α and GCN2 phosphorylation in the same assay (38) (Fig. 3*D*). This assay was ATP and input material dependent (Fig. 3*E*). GCN2 kinase inhibitors blocked GCN2-dependent eIF2α phosphorylation and this effect correlated with the potency of the drug (GCN2-IN-6 was the more potent GCN2i, Table S1 and Fig. 3*F*). By contrast, even at 0.5 μM, torin-1, torin-2, sapanisertib, or rapamycin had no effect on GCN2 or eIF2α phosphorylation (Fig. 3*F*). Taken together, these results show that mTORi lack direct activity toward GCN2 and *vice versa*.

### Torin modulation of GCN2 is independent of eIF2α or PERK

**Figure 4:**
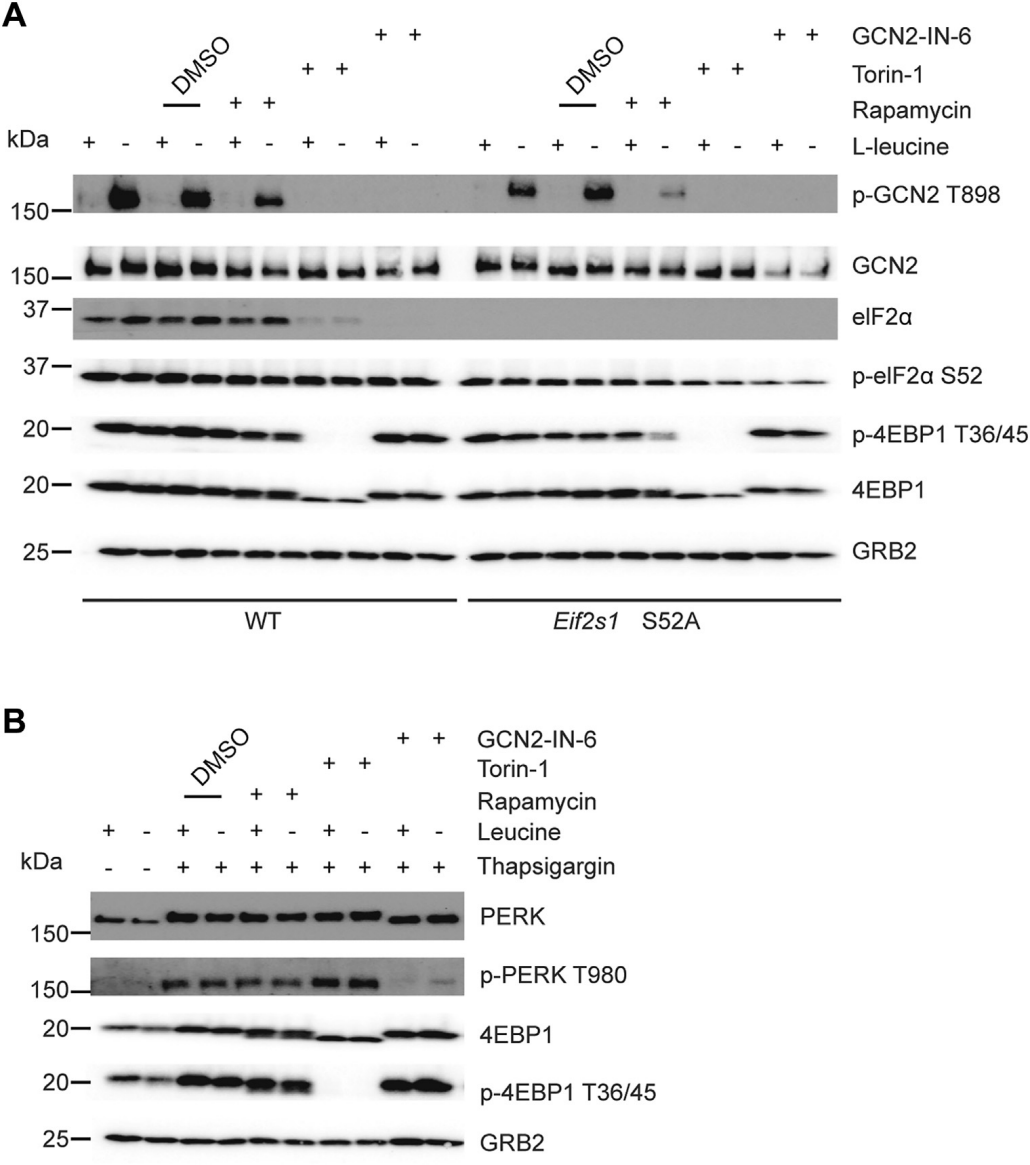
**Role of eIF2α S52 and PERK**. *A*, pathway immunoblotting analysis in mutant eIF2α cells. 3T3 WT cells or 3T3 cells with a biallelic point mutation at serine 52 to alanine (*Eif2s1* S52A) were cultured in media with Leu (+) or lacking Leu (−) for 4 h without or with GCN2-IN-6 (10 μM), torin-1 (1 μM) rapamycin (1 μM) coincident with amino acid starvation. Data are depicted as representative one of two independent experiments. *B*, pathway immunoblotting analysis for GCN2- *versus* PERK-ISR specificity. 3T3 cells were cultured in media with Leu (Ctrl) or lacking Leu (-) for 4 h in the absence or presence of 1 μM thapsigargin. Cells were treated additionally with exogenous leucine, GCN2-IN-6 (10 μM), rapamycin (1 μM), or torin-1 (1 μM). PERK activation was detected with a polyclonal antibody to T980, required for kinase activation or an antibody to total PERK, which detects the mobility shift associated with PERK activation. Note that torin-1 does not inhibit PERK activation, controlled by the inhibition of p-4EBP1 T36/45, while GCN2-IN-6 inhibits PERK activation. Data are depicted as one of two independent experiments. ISR, integrated stress response.

We next considered the mechanistic basis of how torins could modulate the GCN2 pathway. We first tested the proposed role of p-eIF2α in feedback loop regulating mTORC1 (58); in this model, p-eIF2α acts “upstream” of mTORC1 under conditions of amino acid restriction (asparagine and glutamine) by an unknown mechanism. We created 3T3 cells with a biallelic eIF2α knock-in mutation at serine 52 to an alanine (*Eif2s1* S52A), which eliminates the ability of all branches of the ISR to negatively regulate eIF2α while leaving the upstream activation pathways to the ISR intact (59). In eIF2α mutant or WT control cells, torin-1 blocked GCN2 (p-GCN2 T898) and mTORC1 activity (p-S6K T389 and p-4EBP1 T36/45) (Fig. 4*A*), ruling out an obvious p-eIF2α-dependent pathway that connects mTORC1 and GCN2 upon Leu starvation in our system.

To confirm this finding in a different way, we performed a signaling ‘gain-of-function’ experiment to induce p-eIF2α by activating PERK (p-PERK T980) *via* Tg-induced ER stress. Tg triggers the PERK-ISR independent of GCN2 and amino acid stress (Fig. 4*B*). Indeed, Tg strongly induced p-eIF2α and ATF4 expression in the presence of torin-1, regardless of whether the cells were Leu starved. However, torin-1 blocked p-GCN2 T898, while p-PERK T980 was unaffected, indicative for the specificity of torin-1 to affect the GCN2 pathway but not the PERK arm of the ISR. We noted that GCN2-IN-6 inhibited p-PERK T980 (Fig. 4*B*), consistent with reported PERK inhibitory capacity (IC_50_ 0.26 nM) (44) and the high sequence similarity of the PERK and GCN2 kinase domains (Fig. S1*E*).

### Torins block GCN2 in a time-dependent way

**Figure 5:**
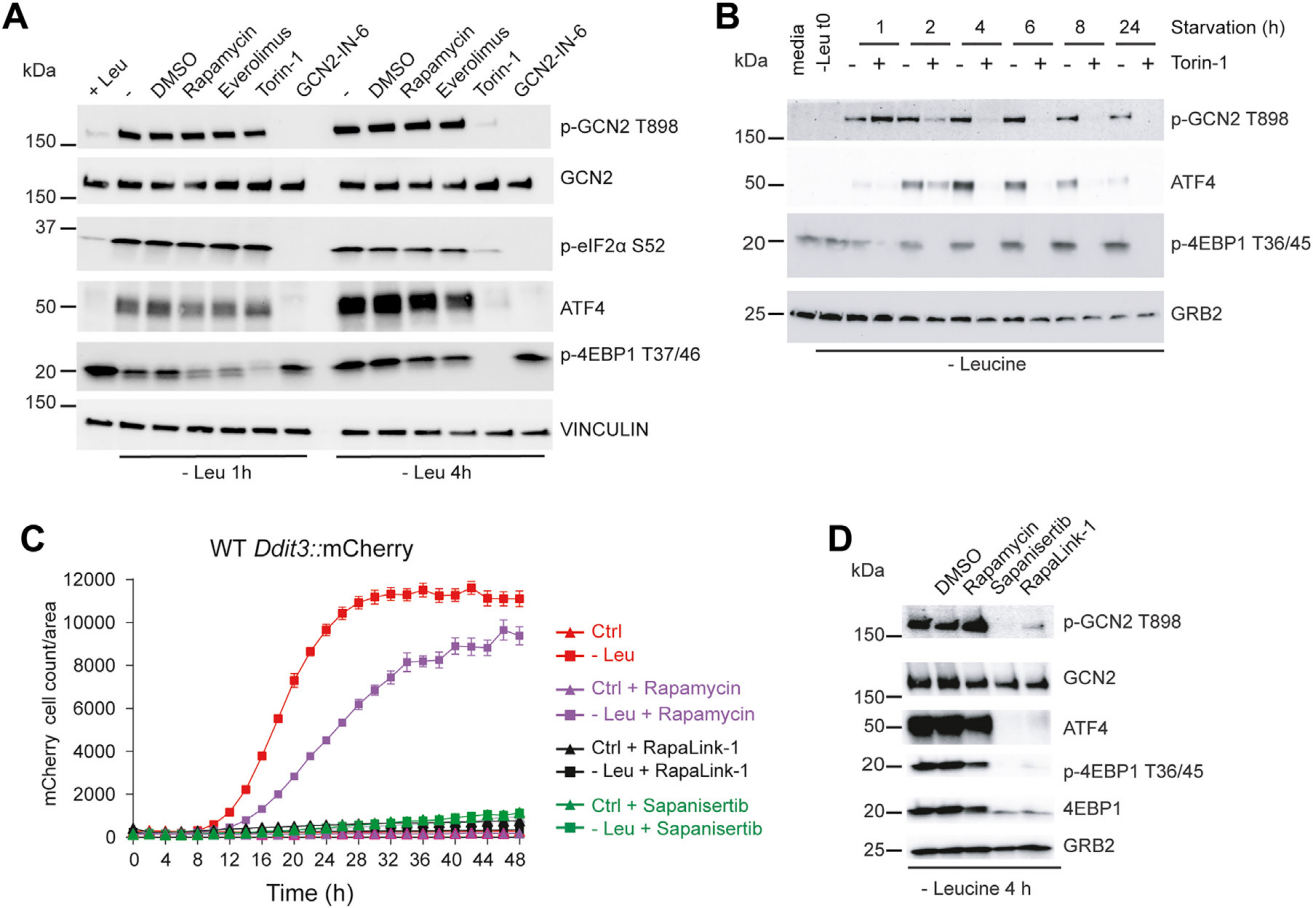
**mTORC1 regulates GCN2 activity postactivation.***A*, time-dependent GCN2 pathway analysis of torin-1 on GCN2 activation. 3T3 cells were starved for Leu for 1 h or 4 h in the absence or presence of GCN2-IN-6 (10 μM), rapamycin (1 μM), everolimus, (1 μM), and torin-1 (1 μM). Lysates were probed for activation of GCN2 (p-GCN2 T898), total GCN2, ATF4 as a marker of the ISR or p-4EBP1. GRB2 was used as a loading control. Note that torin-1 does not inhibit p-GCN2 or ATF4 at 1 h. *B*, 3T3 cells were cultured in Leu-free media for the times indicated in the absence of presence of torin-1 (100 nM). The GRB2 loading control indicates the expected time-dependent decline in total protein amounts in the lysates over the 24 h starvation period. *C*, quantification of the inhibitory effect of RapaLink-1 (10 μM) in *Ddti3*::mCherry 3T3 cells upon L-leu starvation compared with rapamycin (1 μM) and sapanisertib (10 μM). Data are depicted as mean ± SEM of three technical replicates and represent one of two independent experiments. *D*, pathway immunoblotting analysis of the effects of RapaLink-1 compared to its parent inhibitors (rapamycin and sapanisertib) using Leu-starved 3T3 cells for 4 h. Data are representative of three independent experiments. ISR, integrated stress response.

So far, our experiments using torins to block GCN2 activation were performed with 4 h of Leu starvation (or later in the case of the original screen). To investigate if time was a factor in the effect of torins on GCN2 activation, we treated cells with GCN2-IN-6, torin-1, or rapamycin for 1 or 4 h. GCN2-IN-6 inhibited p-GCN2 T898 and ATF4 expression at both time points as expected. Surprisingly, however, torin-1 had no effect on either GCN2 activation or ATF4 expression at 1 h (Fig. 5*A*). These results were recapitulated with a longer time course from 1 to 24 h, which indicated p-GCN2-T899 and ATF4 expression were inhibited by torin-1 beginning at 2 h, and this inhibitory effect was sustained through the end of the experiment at 24 h (Fig. 5*B*). Thus, the effect of inhibitory effect of torin-1 required >1 h to reverse GCN2 activation, after amino acid starvation had already activated the GCN2 pathway. While torins block the GCN2 pathway, rapamycin had no effect (Fig. 4*A*). This suggested that either rapamycin was not sufficient to inhibit the key substrate(s) involved or the mTORC2 complex was required for GCN2 inhibition. To resolve this question, we used rapalink (rapalink1), a bifunctional mTORC1 inhibitor consisting of rapamycin fused to sapanisertib (29). Rapalink completely inhibited *Ddit3*::mCherry reporter activity, p-GCN2 T898 and ATF4 expression, suggesting mTORC1 was responsible for GCN2 pathway inhibition (Fig. 5, *C* and *D*).

### Torin-mediated translational inhibition “frees” Leu to reverse GCN2 activation

**Figure 6:**
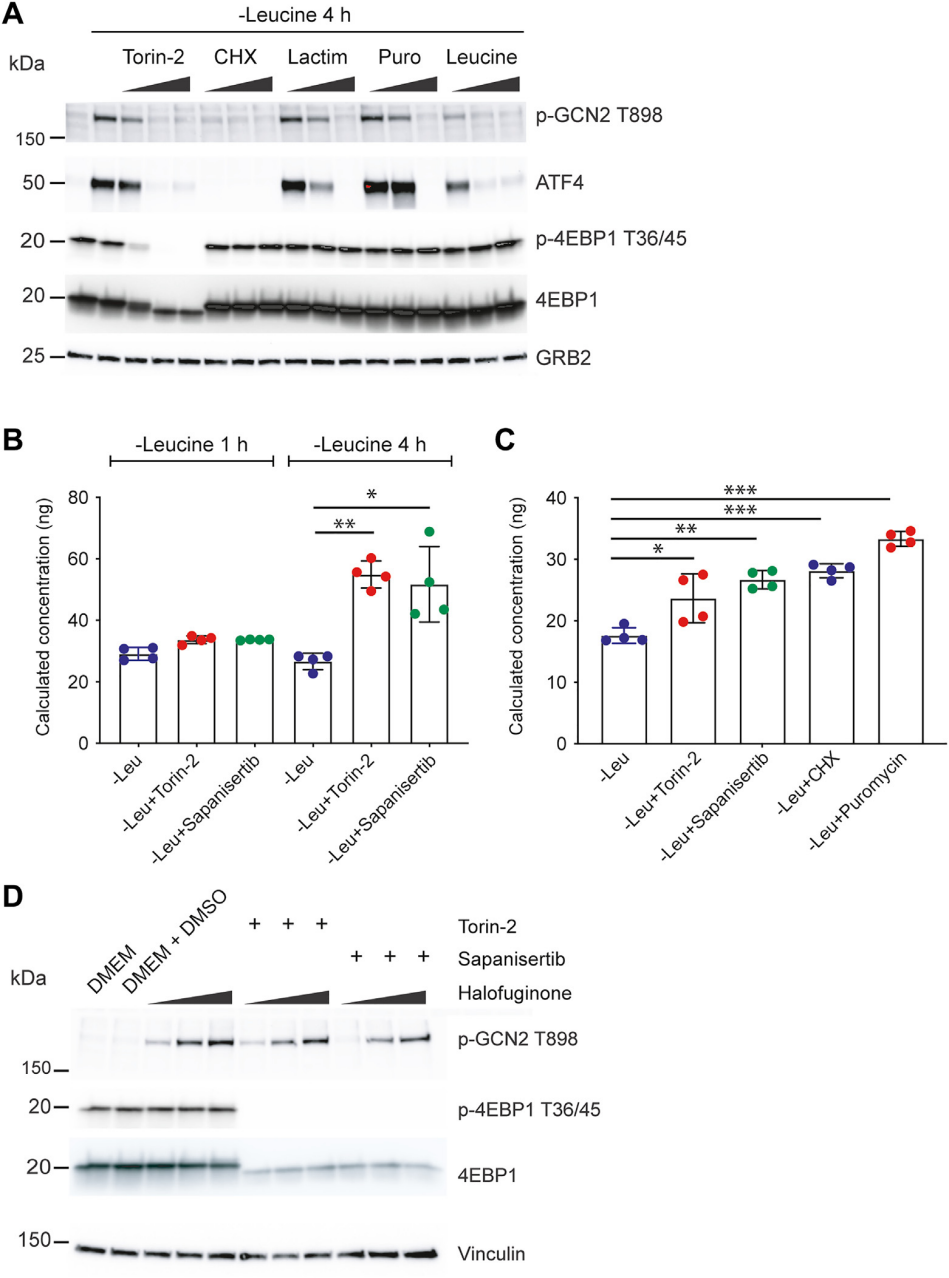
**Translation inhibitors reverse GCN2 activation.***A*, 3T3 cells were cultured in normal media (single left lane) or media lacking Leu for 4 h to induce GCN2 activation. At time zero, torin-2, cycloheximide (CHX), lactimidomycin (Lactim), puromycin (Puro), or leucine was added in the concentrations detailed in the Experimental procedures. Lysates were probed for activation of GCN2 (p-GCN2 T898), ATF4 as a marker of the ISR or p-4EBP1 T36/45 as an indicator of mTORC1 activity. GRB2 was used as a loading control. Note that translation inhibitors do not affect mTORC1 activity and have a differential effect of ATF4 (which is newly transcribed and translated after GCN2 activation). Data are representative of three independent experiments. *B* and *C*, quantification of intracellular Leu by LC-MS. 3T3 cells were cultured as indicated and washed with PBS prior to lysis in acetonitrile/methanol. Experiments were performed using cell samples cultured and processed independently (n = 4). Pairwise comparisons between experimental samples were made by *t* test in Prism. *D*, effect of torin-2 or sapansertib on halofuginone-mediated GCN2 activation. 3T3 cells were cultured in normal DMEM media (which lacks proline) in the presence of halofuginone with or without torin-2 or sapanisertib (100 nM each). Lysates were probed for activation of GCN2 (p-GCN2 T898) or mTORC1 activity using p-4EBP1 T36/45. GRB2 was used as a loading control. Data are presentative of two independent experiments.

A key clue to the mode of action of GCN2 inhibition by torins came from the time-dependency effect described previously. Although torins rapidly block mTOR activity, the fact that ∼2 h were needed to observe an effect on GCN2 activation, raised the possibility that a distinct cellular process was necessary to reverse GCN2 activity. By virtue of their effect on two key substrates involved in translation rate and control (p-S6K and p-4EBPs), mTORC1 controls protein biosynthesis. Therefore, we hypothesized torin-mediated inhibition of mTORC1 may reduce residual protein translation that occurs during amino acid stress and thus allows a reserve of Leu to be available to suppress GCN2 activity; more specifically, the total amount of uncharged tRNAs would decline as mTORC1 translation ceased, suppressing GCN2 activation. We tested this hypothesis in different ways. First, we compared torin-2 with three different inhibitors of translation that act at different points in translation: cycloheximide (CHX), which blocks translation by interacting with the E site of the ribosome, lactimidomycin (60), which inhibits the loading of nascent mRNAs into translating ribosomes, and puromycin, which corrupts peptide bond synthesis by acting as a tyrosyl-tRNA mimetic. Although we note each of these drugs involves complex interactions with the translational machinery and are yet to be fully understood, they all produced similar outcomes in terms of GCN2 activity; they all suppressed GCN2 activation when cells were starved of Leu in a concentration-dependent way (Fig. 6*A*). Notably, CHX completely reversed GCN2 activation without affecting mTORC1 activity toward p-4EBP1 (ATF4 was not detected as CHX blocks new protein synthesis). Therefore, we concluded that translational inhibition was consistent with a model where Leu amounts were available to reverse GCN2 activation. Similar results were obtained in HeLa cells (Fig. S5) noting that these cells manifest baseline p-GCN2 T899 (that is further increased by Leu starvation) and higher concentrations of torins are necessary to reverse the activated p-GCN2.

We next used liquid chromatography-mass spectrometry-based Leu quantification to determine if Leu amounts changed when torins were added to Leu-starved cells where the baseline measurements of the total amount of Leu retained in 3T3 cells after washing was ∼200 ng as a fraction of the input sample (see Experimental procedures)

(Fig. S6, *A* and *B*). Notably and in keeping with their dependence on abundant Leu, HeLa cells had five times greater internal leucine than 3T3 cells (Fig. S6*B*). At 1 h of Leu starvation, when GCN2 activity is robust (Fig. 6*B*), torin-2 or sapanisertib had no significant effect on intracellular Leu amounts. By contrast, at 4 h of starvation, torin-2 or sapanisertib caused an approximate doubling of intracellular Leu. These results were substantiated by comparing the effect of torins to CHX or puromycin, both of which also caused an increase in intracellular Leu (Fig. 6*C*).

### Torins fail to block GCN2 activation when a tRNA synthase is inhibited

Halofuginone is an inhibitor of the prolyl-tRNA synthase component of the EPRS tRNA synthase complex (61). Halofuginone activates GCN2, presumably *via* the increased abundance of deacylated proline tRNAs. We reasoned halofuginone activation should be refractory to torin-mediated reversal of GCN2 because the continued presence of deacylated tRNAs would be sufficient to activate GCN2. We tested this concept by treated 3T3 cells with halofuginone in complete Dulbecco's modified Eagle's medium (DMEM) (with dialyzed serum), which lacks proline. Cell culture in DMEM, therefore, forces cells to rely on endogenous proline biosynthesis and proline recycling. We found halofuginone caused a concentration-dependent GCN2 activation that was not reversed by either torin-2 or sapanisertib, consistent with the concept that torin-mediated inhibition of mTORC1 cannot suppress GCN2 activation in the continuous presence of uncharged tRNAs (Fig. 6*D*).

## Discussion

The rationale for developing GCN2 inhibitors centers on two principlesthat (i) GCN2 is non-essential in normal cells and animals, which conceivably reduces the risk of drug toxicity, and (ii) that malignant cells have increased cell-specific dependencies on amino acids to sustain their proliferation, raising the likelihood that some cancerous cells depend on GCN2 and ISR signaling to temper the rate of translation. To this end, different types of GCN2 inhibitors have been identified. In our hands, several of these inhibitors have limitations that include toxicity (GZD824) or promiscuous inhibitory activity to PERK (GCN2-IN-6), which is an essential ISR kinase, to weak GCN2 inhibitory activity in the micromolar range. We therefore developed a cell-based screen to search for new GCN2 inhibitors by harnessing the expression profile of CHOP under amino acid limitation. CHOP expression is induced by limiting amino acids in a GCN2-dependent way and is reversible by resupply of amino acids. Although CHOP is also induced by all ISR kinases, we designed a way of ascertaining GCN2 dependency of any isolated GCN2i through the use of isogenic matched CHOP reporter lines on GCN2-deficient backgrounds combined with signaling pathway dissection. The net result of screening for inhibitors of CHOP expression in leucine-free media resulted in the isolation of numerous mTOR and PIKK family ATP competitive inhibitors (torins).

Our results showed torins inhibited GCN2 in a concentration-dependent way (in the nM range) *via* an indirect mechanism that involves translational suppression. The simplest interpretation of our results is that under complete Leu starvation, GCN2 is activated by the combination of an increased threshold amount of uncharged tRNAs, in combination with GCN1-mediated recognition of stalled ribosomes. At the same time, we speculate that mTORC1-mediated translation continues but at a reduced rate due to the limitation of leucine. Once torins inhibit mTORC1, translation is further lowered (although, importantly, not for GCN2 ISR targets that are translated such as ATF4 and CHOP). In this model, sufficient leucine may be made available to reacylate uncharged leucine tRNAs and reverse GCN2 activity. This model is predicated on three experimental findings: (i) the suppression of GCN2 activity by torins is strongly time dependent and only begins ∼2 h after GCN2 is activated by leucine starvation. In this setting, GCN2 activation is rapidly activated by uncharged leucine tRNAs, while mTORC1 is inhibited. Over time, the decline in translational activity mediated mTORC1 inhibition may be sufficient to mobilize enough leucine to reverse GCN2 signaling. In line with this idea, GCN2 activity in the presence of torins is sustained over 24 h, suggesting that cells in this situation (GCN2 and mTORC1 “off”) are in a state of “hibernation.” (ii) General translational inhibitors, like torins, also reverse GCN2 activity during leucine starvation (see Limitations of the study, later), and (iii) torins and translational inhibitors increase the amount of leucine inside cells compared to leucine starved cells without torin or translational inhibitor treatment. Taken together, these lines of experimentation support a model where GCN2 signaling is controlled in a switch-like way that is dependent on a threshold amount of leucine (or any limiting amino acid) and charged leucine tRNAs. Notably, torin-1 inhibits ATF4 and CHOP expression in RAS-transformed murine embryonic fibroblasts lines (p-GCN2 was not reported in these experiments) that are dependent on harvesting extracellular protein, and under these conditions, cell death was prevented by mTOR inhibition (20). These results parallel our findings in a different system and suggest that GCN2 and mTORC1 have a dynamic and complex interrelationship during amino acid starvation.

Hinnebusch *et. al.* originally proposed that GCN2’s histidyl tRNA synthase–like domain bound uncharged tRNAs, leading to dimerization, autophosphorylation, and kinase activity toward eIF2α (40). Subsequently, other components of the GCN2 apparatus have been uncovered including the heat repeat protein GCN1 (62), which in yeast seems to interact mainly with the 40S subunit collided ribosomes (63) and the ribosomal P-stalk. A major gap in understanding how each of these components works together to activate GCN2, and then how GCN2 ultimately mediates ISR signaling remains incomplete. When considered in the light of our current work, we argue that GCN2 activation may depend on all the aforementioned components working in unison; under amino acid restriction (*i.e*., when leucine can only be derived from intracellular stores or existing proteins), the probability of ribosome stalling increases as the number of uncharged leucine tRNAs also increases. GCN1 is recruited to the stalled ribosomes and aids in recruiting GCN2, which detects an uncharged leucine tRNA at the P site, leading to dimerization and activation. This model, although speculative and requiring high resolution structural information, suggests that GCN2 is activated at stalled ribosomes (39, 62, 63, 64). Once sufficient charged tRNAs are available and enough collided ribosomes resolved, GCN2 activation is reversed.

We used halofuginone to test the concept that torins would not be able to reverse GCN2 activation once the proline tRNA synthase (EPRS) was inhibited. Such an outcome was indeed the case as neither torin-2 nor sapanisertib, the strongest ‘reversers’ of GCN2 activation, could not suppress GCN2 activation by halofuginone. We interpret these results in the following way: halofuginone blocks EPRS and causes a rise in uncharged proline tRNAs that ‘saturate’ the GCN2 pathway. Torins cannot overcome the effect of halofuginone under these conditions because uncharged tRNAs are continuously generated. We note that other groups have recently uncovered different aspects of halofuginone activity including GCN2-independent but GCN1-dependent effects, suggesting that a complete understanding of the mechanism of action of this drug is so far unclear (61, 65, 66, 67). In our cell system, however, we exclusively used DMEM or DMEM Stable Isotope Labeling by/with Amino acids in Cell culture media, which lacks proline. 3T3 cells must, therefore, synthesize, recycle, or scavenge proline, which may enhance the ability of halofuginone to activate GCN2.

Our experiments, and those of others, point to layered communication between GCN2 and mTORC1-regulated translational control. Indeed, numerous studies have observed evidence for an interplay between these pathways, which can only occur when amino acids are limiting and GCN2 activated. In our experimental setting of leucine-free media, mTORC1 competes with GCN2-activated ISR translation, even though the overall translation rate must be much less than the rate in normal media. When the “residual” mTORC1-mediated translation is blocked by torins, GCN2 is suppressed. But does this work in the other direction? Here, there is less information except for the finding that GCN2-deficient cells have increased mTORC1 activity when starved of different amino acids (21, 46, 58, 68, 69). These types of experiments highlight how little we understand the dynamics of where amino acids are stored in the cell, the overall ribosome dynamics at individual and population levels, and how the “upstream” amino acid–sensitive kinase ultimately contribute to ribosome translational efficiency and control. Finally, we note that torins are pervasively in experimental biology used as a chemical tool to block mTOR activity; our findings suggest that torins will block GCN2 activity under conditions involving amino acid starvation that is intentional (*i.e*., deprivation of an amino acid from media or by an enzyme that degrades a key amino acid such as asparaginase) or unintentional when cells naturally consume available amino acids, forcing the activation of GCN2 signaling. Therefore, circumspection in the interpretation of the effect of torins should account for possible perturbation of GCN2 pathway, in addition to mTOR inhibition (70).

### Limitations of the study

Our cell-based study has three limitations. First, the full mechanistic scope of how different translational inhibitors work in the context of cellular translation remains unclear (60, 71). For example, puromycin does not simply block nascent chain elongation but also rapidly dissociates the large and small ribosome subunits (71). In our model, stalled ribosomes are central to GCN2 activation and should puromycin cause ribosome dissociated, the GCN2 activity would be suppressed independent of amino acid amounts. Second, the exact mechanism of GCN2 activation requires structural illumination combined with a knowledge of the dynamic relationship between amounts of amino acids required in different cellular compartments; different cells have substantially different ways of harvesting or mobilizing amino acids depending on their needs (16, 19, 20, 72). Such pathways have been uncovered especially in different cancer cell types. Third, we cannot exclude contributions to GCN2 signaling reversal from different autophagy and proteosome-mediated amino acid supply in our system since three simultaneous manipulations are needed: leucine starvation (inducing autophagy), torin treatment (which also induces autophagy), and an autophagy or proteosome inhibitor. We consider such combinations of variables that simultaneously induce and inhibit pathways difficult to interpret without precise genetic control over each component. Thus, future studies will need to incorporate genetic lesions in these pathways, such as an ATG5- or ATG7-deficient system.

## Experimental procedures

### Cell culture, specialized media, and drugs

NIH-3T3 cells (3T3; CCL-92) and HeLa cells (CCL-2) were purchased from the American Type Culture Collection (ATCC), cultured, and maintained in DMEM (41966-029, Thermo Fisher) supplemented with 10% fetal bovine serum (10270, Life Technologies) and 1% penicillin–streptomycin (P/S; 09-757F, Lonza) in humidified tissue culture incubators at 37 °C with 5% CO_2_. Cells were not used above passage 5 and tested for *mycoplasma* infection by PCR screening (LookOut *mycoplasma* PCR detection kit, MP0035-1KT, Sigma–Aldrich). Starvation medium contained DMEM (9443, Sigma–Aldrich, lacking L-arginine, L-leucine, and L-lysine) supplemented with homemade 5% dialyzed fetal bovine serum, 3.5 g/l glucose (A2494001, Gibco), and 1% penicillin–streptomycin (09-757F, Lonza). Depending on the amino acid depletion, the medium was supplemented with additional Arg HCl (0.084 g/l;1689.3, Carl Roth), Leu (0.105 g/l;1699.1, Roth), and/or Lys HCl (0.146 g/l; 1700.1, Carl Roth). Amino acid stocks were prepared in PBS (10010015, Life Technologies). Amino acid starvation was induced by washing 60% to 70% confluent adherent cells (plated at 2.5 × 10^5^ cells per well in day before in 1 ml complete DMEM) three times with 1× PBS (10010015, Life Technologies) and adding amino acid starvation medium according to the appropriate volume indicated by the used tissue culture plate format (in general 12-well format was used in most experiments). Drug treatment was performed at the same time as amino acid starvation and used concentration is indicated in the respective figure legend. Drugs were made in dimethyl sulfoxide (DMSO) (12611S, CST) and purchased if not supplied by Dr Jan Eickhoff: puromycin (P8833, Sigma–Aldrich), rapamycin (553210, Calbiochem), Tg (586005, Merck), torin-1 (14379S, CST), torin-2 (SML1224, Sigma–Aldrich), BAY1895344 (HY-101566, MCE), GCN2iB (HY-112654, MCE), berzosertib (M6620, Sigma), RapaLink-1 (HY-111373, MCE), and everolimus (HY-10218, MCE).

### Ddit3 reporter cell line construction and validation

Three partly overlapping guide RNAs (oligonucleotides and repair plasmids) targeting the initiation codon region of *Ddit3* (exon 2) were cloned into pX330-GFP (a first generation Cas9 vector from Feng Zhang’s laboratory, no longer available from Addgene and replaced by pSpCas9(BB)-2A-GFP (PX458); 42230 Addgene). Plasmids were transfected into 3T3 cells using Lipofectamine 3000 (L3000015, Thermo Fisher) along with a synthetic ‘repair’ plasmid consisting of mCherry coupled to the SV40 polyadenylation sequence and ∼200 nt flanking sequence on either side of exon 2 of *Ddit3*. Successful homologous recombination replaced the entire coding exon of *Ddit3* with mCherry translation beginning with the exact genomic and transcript context as *Ddit3*. A similar approach, using the same guides, was used to construct the NanoLuc-PEST reporter, which was cotransfected with a GFP-expressing plasmid for sorting. Following sorting for GFP^+^ cells, a fraction of the ‘bulk’ population was assessed by PCR for the presence of the recombined alleles using primer combinations outside the flanking arms. Single cell cloning in 96-well plates was then performed to isolate individual clones, which were next expanded and duplicated. Each clone was incubated in normal DMEM or DMEM lacking leucine for 24 h and mCherry expression assessed by IncuCyte S3 live-cell imaging (IncuCyte S3, EssenBioscience). Single clones were selected based on the criteria of low mCherry background in normal media and high mCherry signal in media lacking leucine. A similar procedure was performed for the NanoLuc-PEST construct reading out chemiluminescence upon L-leu stress in a Tecan plate reader (Infinite 200 PRO, Tecan). As confirmed by Sanger sequencing, *Ddit3* was mutated at one allele and did not affect *Ddit3* expression from the other allele.

### Mutation of eIF2α

The codon encoding serine 52 of *Eifs1* (encoding eIF2α, numbering follows the mouse protein, which is equivalent to serine 51 in most other species) was modified to encode an alanine reside by using CRISPR/Cas9 genome engineering. Two guides were designed close to the region encoding serine 52 (oligonucleotides and repair plasmids), cloned into pX330-GFP, and cotransfected into 3T3 cells with a synthetic 1400 bp repair construct containing the targeted mutation and multiple mutations to ablate further Cas9 activity at the repaired locus. GFP^+^ cells were sorted and cloned to single cells and then assessed by competitive real-time PCR for the mutated codon. Correctly recombined clones were then expanded and checked for eIF2α S52 phosphorylation using a specific antibody to this phosphosite (described later).

### Live-cell imaging and luciferase reporter analysis

mCherry induction was monitored by live-cell phase-contrast microscopy (IncuCyte S3, EssenBioscience) over time by reading out the red fluorometric channel (red object intensity/image). The *Ddit3*::mCherry cells and/or their GCN2-deficient counterpart were seeded in 96-well (92696TPP, TPP), 48-well (3548, Corning), or 12-well tissue culture plate format (3513, Corning) to reach a 60% confluency on the next day. The cells were treated according to their experimental purpose. 3T3 cells were used as negative control for the assay background. Images were taken at 10× objective with an acquisition time of 400 ms and a mask was applied to each image overlaying mCherry^+^ to mCherry^-^ cells. mCherry^+^ cells were normalized to cellular confluence (red object count per image/phase area confluence (1/Image/%)). Nano-Luciferase induction was tracked by measuring the luminescence with an integration time of 1 s in a Tecan plate reader (Infinite 200 PRO, Tecan). The assay procedure is based on the manufacturer’s instructions using the Nano-Glo Dual Luciferase reporter assay kit (N1610, Promega). The cellular luminescence intensity was monitored in white opaque 96 flat bottom tissue culture plates (353296; Falcon). The 3T3 *Ddit3*::NanoLuc-PEST cells were seeded in triplicates per treatment condition in a 96-well tissue culture format (92696, TPP) to reach a 60% confluency on the next day. The cells were treated according to their experimental purpose. 3T3 control cells were used to control for the luciferase assay background signal.

### Cell death detection assay

3T3 cells were seeded to 60% confluency in tissue culture plates (12-well format) and treated with CellTox Green reagent (1:4000, G8741, Promega) the next day. Cell death was monitored by live-cell phase-contrast microscopy over time by reading out the green fluorometric channel. Images were taken at 10× objective with an acquisition time of 300 ms and a mask was applied to each image and normalized to cellular confluence (green object count per image/phase area confluence (1/Image/%)). The analysis parameters were identical for all groups and time points.

### Immunoblotting

Cells were lysed in radioimmunoprecipitation assay lysis buffer supplemented with a protease and phosphatase inhibitor cocktail (100×; 78440, Thermo Scientific). Cell lysates were separated on 4% to 15% Criterion TGX Stain-Free protein gels (5678085 or 5678084, Bio-Rad) using Bio-Rad Precision Plus markers (#161-0394). Following transfer to nitrocellulose membranes (0.2 μm, 10600001, Amersham) and blocked in 3% nonfat milk (170-6404, Roth) or 1% to 3% bovine serum albumin (BSA) (A2059; Sigma–Aldrich) in Tris-buffered saline containing 0.1% Tween-20. Membranes were cut according the molecular weight of the target proteins, which were validated by genetically deficient cell lysates (GCN2-deficient cells), the known and predictable pattern of expression (ATF4, CHOP) or known behavior of protein phosphorylation (*e*.*g*., inhibition of p-4EBP1 by torins). In some cases, membranes were cut to remove crossreactive nonspecific proteins (anti-p-GCN2 T899, which has highly crossreactive bands at ∼220–280 kDa). Membranes were incubated overnight at 4 °C with the following primary antibodies in blocking buffer: PERK (C33E10) (1:2000; 3192, Cell Signaling Technology, CST), p-PERK T980 (16F8) (1:300; 3179, CST), GCN2 (1:800; 3302, CST), p-GCN2 T899 (1:1000; ab75836, Abcam), eIF2α (D7D3) XP (1:1000; 5324, CST), p-eIF2α S51 (119A11) (1:1000; 3597, CST), GRB2 (1:1000; 610112, Becton Dickinson), 4EBP1 (53H11) (1:1000; 9644, CST), p-4EBP1 T37/46 (236B4) (1:1000; 2855, CST), mCherry (1:2000; ab167453, Abcam), Akt (pan) (C67E7) (1:1000; 4691, CST), p-Akt S473 (1:1000; D9E9 XR, 4060, CST), Vinculin (1:1000; 13901, CST), ATF4 (1:1000, sc-390063, Santa Cruz), and CHOP L63F7 (1:500; 2895, CST). Membranes were washed in 1×TBST and water before incubation with 1:10,000 horseradish peroxidase–conjugated secondary antibodies goat anti-rabbit (111-035-003, Jackson ImmunoResearch) and goat anti-mouse (115-035-003, Jackson ImmunoResearch) before visualization with SuperSignal West Pico Substrate (34080; Pierce). Visualization of the protein was monitored in the ChemiDoc Imager (Bio-Rad). Separate, parallel blots were performed because of the size overlap of protein detection. GRB2 and Vinculin were used as loading controls depending on the other targets.

### Compound screening and validation

A library of 3876 compounds enriched with published kinase inhibitors was screened at 2 μM or 10 μM concentration for inhibition of leucine starvation-induced CHOP activation in *Ddit3*::NanoLuc-PEST cells. Cells were seeded in poly-D-Lysine–coated 384 cell culture grade assay plates (781945, Greiner Bio-One GmbH) at 7000 cells/well in 25 μl of culture medium. Twenty-four hours after seeding, wells were washed with PBS followed by addition of starvation medium or medium supplemented with all amino acids for the nonstarved control wells. Directly after the medium exchange, test compounds or DMSO were added to the cells, followed by incubation for 28 h and measurement of luciferase activity. Hits with known cytotoxic activity or undesired properties of chemical structures were removed. Filtered hits displaying >50% inhibition at the tested concentration were validated in 8-point 3-fold response curves starting from 10 μM.

### IP and kinase assays

3T3 cells were transiently transfected with GCN2-3xFlag plasmid (101794, Addgene), L-leu-starved for indicated times shown in the figure legends, lysed in Triton-X 100 containing buffer (50 mM Tris pH 7.4, 150 mM NaCl, and 1 mM EDTA) supplemented with protease and phosphatase inhibitors (100×; 78440, Thermo Scientific) and transferred to prechilled tubes. Then, anti-Flag M2-affinity gel beads (A2220 Sigma; 8 μl/200 μl lysate) were added and incubated for 1 h at 4 °C on a moving rotor. The supernatant was carefully removed after centrifugation at 12,750 rpm for 1 min and washed with 1 ml lysis buffer without Triton-X 100. Then, 0.5 mg/ml 3×-Flag peptide (MPI-Biochemistry Core Facility) was added per sample, incubated for 15 min with occasional agitation, and eluted in wash buffer for a final volume of 10 μl. The reaction conditions were modified from Harding *et. al.* (38) contained 0.5 mg/ml BSA, 1 M DTT, 1 M β-glycerophosphate, 0.5 mM ATP, 1 M MgCl_2_, 294 μM recombinant human eIF2α, and 10 μl of 2× reaction buffer (25 mM Hepes pH 7.4; 50 mM potassium acetate, 2.5 mM magnesium acetate) and added to the 10 μl IP sample, transferred to BSA-coated (100 mg/ml BSA previously coated for 2 h) tubes and incubated at 32 °C in a PCR cycler (Bio-rad) for 10 min. The reaction was quenched by adding 5 μl of 94 °C prewarmed 6× SDS sample buffer and stored at -80 °C for immunoblotting. Whole cell lysates and reactions omitting ATP were used as internal controls for enzyme activity. The GCN2 and mTOR inhibitors were added either to the eluate or during the starvation period. *In vitro* mTOR activity was assayed in 12 μl reactions containing the kinase mTOR^ΔN^-LST8 (100 nM) and the substrate GST-AKT1 450 to 480 (5 μM) in a specific reaction buffer (10 mM MgCl_2_, 1 mM DTT in PBS) (51). Before, the inhibitors were added to the kinase in different concentrations (200 nM, 100 nM, and 10 nM) and incubated for 10 min on ice. To start the reaction, radiolabeled ATP mix (0.5 mM ATP, 0.8 μCi [γ-^32^P]-ATP) was supplied for 30 min at 30 °C. Afterward, the reaction was quenched by adding 2× SDS sample buffer for 2 min at 55 °C and proteins Coomassie stained (12.5% SDS gel) and detected by autoradiography using an Amersham Typhoon RGB imager (Cytiva). As internal controls, the reaction was performed with ATP mix with the addition of 1% DMSO to mimic inhibitor treatment. The negative control was performed without mTOR^ΔN^-LST8 and the positive control with mTOR^ΔN^-LST8 addition.

### Leucine quantification

LC-MS/MS analysis was performed on a QExactive HF mass spectrometer (Thermo Scientific) coupled to a Dionex Ultimate 3000. Cells were lysed in MeOH/ACH/water (2:2:1, v/v) by repetitive freeze thawing in liquid nitrogen, followed by 10 min centrifugation to remove debris. Vacuum-dried metabolite extracts were dissolved in 30 μl of buffer A (0.1% formic acid) and 4 μl of the samples were injected and separated on a C18 column (2.1 × 100 mm, 1.6 μm, LunaOmega Polar C18, Phenomenex) using a gradient of buffer A to 10% buffer B (0.1% formic acid, 80% acetonitrile) in 10 min at a flow rate of 350 μl/min. The mass spectrometer was operated in positive mode with a data-dependent MS1 scan from 90 to 1100 m/z at a resolution of 120k. Sheath gas (N2) flow rate was set to 49 (arbitrary units), auxiliary gas flow rate was 12 (arbitrary units), and sweep gas flow rate was 2 (arbitrary units). The spray voltage was set to 3.20 kV and the capillary temperature to 259 °C. Up to 15 of the top precursors were selected and fragmented using higher energy collisional dissociation (stepped-HCD with normalized collision energies of 10, 20, and 30). The MS2 spectra were recorded at a resolution of 30k. The automatic gain control target for MS1 and MS2 scans were set to 3E6 and 1E5, respectively, within a maximum injection time of 100 and 50 ms for MS and MS2 scans, respectively. Leucine peaks were identified based on MS1 high resolution mass, characteristic MS2 fragments, and retention time using the software “Skyline,” version 21.2.0.425 (MacCoss lab, University of Washington). Quantification of leucine was achieved through extraction of MS1 peak areas of leucine *via* a calibration curve.

### Translational inhibitor experiments

Cells (3T3 or HeLa) were plated at 2.5 × 10^5^ cells per well (12-well) the day before the experiment. Inhibitors were made to a 2× concentration and diluted as shown in the figure legends across a 100-fold range. Cells were then washed twice in PBS to remove the complete media and replaced with 0.5 ml SILAC media lacking leucine. About 0.5 ml of each inhibitor was then added to reach the final 1× concentration and cells incubated for 4 h. Cells were then washed with PBS and lysed in 100 μl radioimmunoprecipitation assay buffer (Abcam). Inhibitors were CHX (50 μg/ml top point), lactimidomycin (MedChem Express, 2 μM top point), and puromycin (10 μg/ml). For the comparison to torins, either torin-1, torin-2, or sapanisertib were used at the concentration dilutions shown in the figure legends.

### Statistical analysis

Graphs were generated using the software GraphPad Prism (version 7.03) (GraphPad Software Inc). All data involving statistics are presented as means ± SEM and the number of replicates mentioned in the figure legends.

### Inhibitors and specialized chemicals

All chemicals, suppliers, and formulations are available in Tables S1 and S2.

### Oligonucleotides and repair plasmids

All oligonucleotide and repair constructs are available in Tables S3–S5.

## Data availability

All source data is available from the corresponding author upon reasonable request.

## Supporting information

This article contains supporting information.

## Conflict of interest

P. J. M. is on the scientific advisory boards of Palleon Pharmaceuticals and ImCheck Pharma, neither of which have activities or interests related to this article. The authors declare that they have no conflicts of interest with the contents of this article.
