# Supplementary material for: A cell-based chemical-genetic screen for amino acid stress response inhibitors reveals torins reverse stress kinase GCN2 signaling
Source: J Biol Chem. 2022 Oct 20;298(12):102629. doi: 10.1016/j.jbc.2022.102629 (PMC9668732; doi:10.1016/j.jbc.2022.102629)
Supplement: Supplemental data [file mmc1.docx]

**Supplemental Material**

**A cell-based chemical-genetic screen for amino acid stress response inhibitors reveals torins reverse stress kinase GCN2 signaling**

Johanna B. Brüggenthies et al.

**Table S1: Compound categorization**

| **Compound** | **Internal**  **Nomenclature** | **Target** | **IC_50_**  ***Ddit3***  **[µM]** | **Part of the primary screen** |
| --- | --- | --- | --- | --- |
| R406 | #1 | Syk/FLT3 | unknown | no |
| Palbociclib | #2 | CDK4/6 | unknown | no |
| Sunitinib | #3 | VEGFR2/PDGFRβ | unknown | no |
| WZ4002 | #4 | EGFR | unknown | no |
| GCN2-IN-1/A-92 | #5 | GCN2 | unknown | no |
| Bosutinib | #6 | Src/Abl | unknown | no |
| Vemurafenib | #7 | B-RAF | unknown | no |
| Lestaurtinib | #8 | JAK2/FLT3/TrkA | unknown | no |
| RAF265 | #9 | RAF/VEGFR2 | unknown | no |
| ML 786 | #10 | RAF | 0.484 | yes |
| PH-797804 | #11 | p38α/p38β | 0.743 | yes |
| Sapanisertib/INK-128 | #12 | mTOR | 0.025 | yes |
| PF 670462 | #13 | CK1ε and CK1δ | 0.485 | yes |
| PIK-75 | #14 | DNA-PK/p110α | 0.0868 | yes |
| GSK2656157 | #15 | PERK | unknown | no |
| p38 Inhibitor | #16 | P38 | 0.874 | yes |
| CDK7 Inhibitor | #17 | CDK7 | 0.15 | yes |
| Omipalisib | #18 | PI3K | 0.09 | yes |
| Tropisetron | #19 | 5-HT3 receptor | 0.37 | yes |
| Amcinonide | #20 | NO release | < 0.008 | yes |
| Mupirocin | #21 | isoleucyl-transfer RNA | 0.0943 | yes |
| Skepinone-L | #22 | p38 | 0.182 | yes |
| ETP-46464 | #23 | mTOR/ATR/ATM | 0.779 | yes |
| WYE-132 | #24 | mTOR | 0.0956 | yes |
| GZD824 | #25 | pan-Bcr-Abl | 0.135 | yes |
| Ralimetinib | #26 | p38 | 0.1 | yes |
| GSK2606414 | #27 | PERK | unknown | no |
| GCN2-IN-6 | #28 | GCN2/PERK | unknown | no |

**Table S2: mTOR and GCN2 key compounds**

| **Compound** | **Synonym** | **Sub-class** | **IC_50_**  **enzymatic** | **Mode of action** | **Structure** |
| --- | --- | --- | --- | --- | --- |
| Sapanisertib | INK-128; MLN0128;  TAK-228 | mTORi | 1 nM | ATP-competitive | 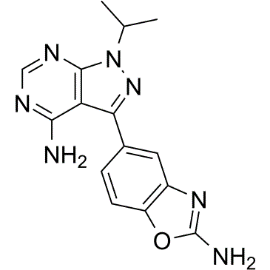 |
| BAY1895344 | Elimusertib | mTORi | 61 nM | ATP-competitive | 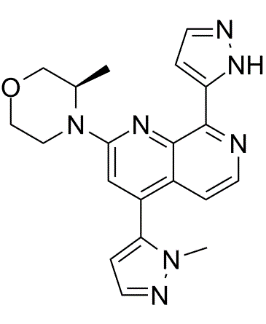 |
| Omipalisib | GSK2126458 | mTORi | 0.18-0.3 nM | ATP-competitive | 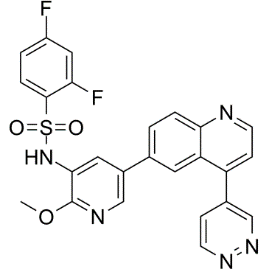 |
| ETP-46464 |  | mTORi | 0.6 nM | ATP-competitive | 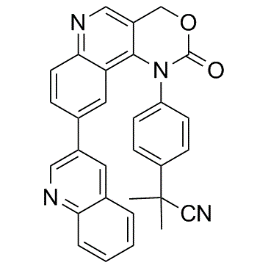 |
| Torin-1 |  | mTORi | 3 nM | ATP-competitive | 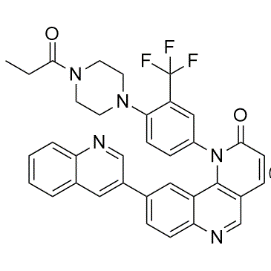 |
| Torin-2 |  | mTORi | 2.81 nM | ATP-competitive | 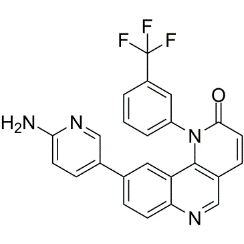 |
| Rapamycin | Sirolimus; AY 22989 | mTORi | 0.1 nM | allosteric | 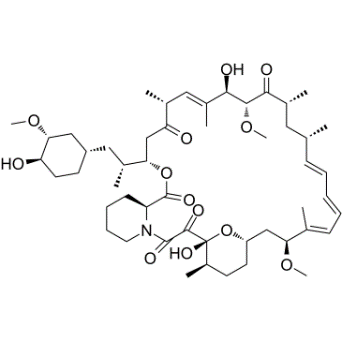 |
| GCN2-IN-1 | A-92 | GCN2i | < 0.3 µM | Allosteric pocket type 1 half inhibitor | 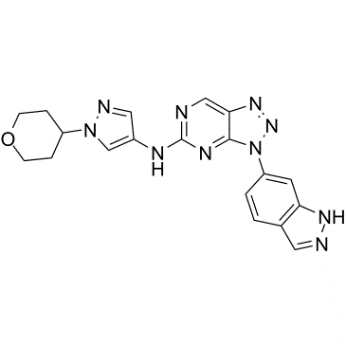 |
| GCN2-IN-6 |  | GCN2i  PERKi | 1.8 nM | Allosteric pocket type 1 half inhibitor | 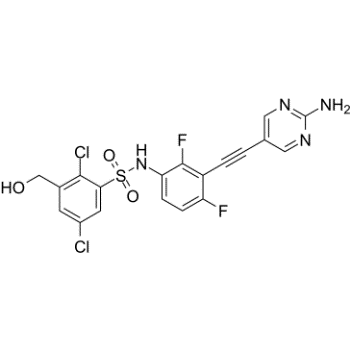 |
| GCN2iB |  | GCN2i | 2.4 nM | ATP-competitive | 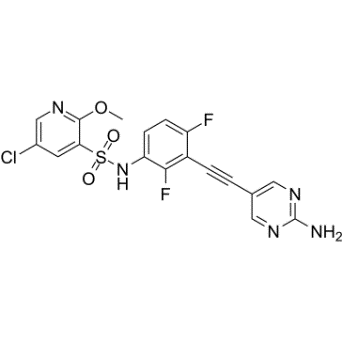 |

**Table S3: Guides to target the 5’ end of the *Eif2s1*; *Eif2ak4* and *Ddit3* coding sequences**

| **5’ to 3’ sequence** | **Nomenclature** |
| --- | --- |
| CACCGGCAAAGTAGCGGACGATATT | Oligo_F_gRNA1_mEif2ak4_exon9 |
| AAACAATATCGTCCGCTACTTTGCC | Oligo_R_gRNA1_mEif2ak4_exon9 |
| CACCGAGTAGCGGACGATATTTGGA | Oligo_F_gRNA2_mEif2ak4_exon9 |
| AAACTCCAAATATCGTCCGCTACTC | Oligo_R_gRNA2_mEif2ak4_exon9 |
| CACCGCTCCATTGTGATCGACATTC | Oligo_F_gRNA3_mEif2ak4_exon9 |
| AAACGAATGTCGATCACAATGGAGC | Oligo_R_gRNA3_mEif2ak4_exon9 |
| CACCGTATAGAACGGATACGTCGTC | Oligo_F_gRNA1_mEif2s1_exon2 |
| AAACGACGACGTATCCGTTCTATAC | Oligo_R_gRNA1_mEif2s1_exon2 |
| CACCgGCCATGACTGCACGTGGACC | Oligo_F_gRNA1_mDdit3_exon3 |
| AAACGGTCCACGTGCAGTCATGGCC | Oligo_R_gRNA1_ mDdit3_exon3 |
| CACCgACCTGGTCCACGTGCAGTCA | Oligo_F_gRNA2_ mDdit3_exon3 |
| AAACTGACTGCACGTGGACCAGGTC | Oligo_R_gRNA2_ mDdit3_exon3 |
| CACCgTCAGCTGCCATGACTGCACG | Oligo_F_gRNA3_mDdit3_exon3 |
| AAACCGTGCAGTCAGTGCAGCTAGC | Oligo_R_gRNA3_mDdit3_exon3 |

**Table S4: Sequencing primers**

| **5’ to 3’ sequence** | **Nomenclature** |
| --- | --- |
| TGAAACACCAGCTAATGTCA | S_Eif2ak4_exon9_F |
| GGAGTTGCTGTGTAGGTAAT | S_Eif2ak4_exon9_R |
| GGGCCTATGTCAGCTTGTTG | S_Eif2s1_exon2_F |
| CTGCCAATTCGGATCAGTTT | S_Eif2s1_exon2_R |
| HEX-CGACGTATCCGTTCTATAAAC-ZEN | WT Eif2s1 hybridization probe |
| FAM-CGCCGCATTCGATCCATTAAC-ZEN | Mutant Eif2s1 hybridization probe |
| TGGAATGTATGTCCTTTCCA | S_Ddit3_exon3_F |
| CTCTTGCCTATACTTGACAA | S_Ddit3_exon3_R |

**Table S5: Repair constructs**

Mouse *Eif2s1* S52A locus modification: repair construct (generated by complete gene synthesis, IDT). Mutations to suppress further Cas9 activity are lower case within the uppercase part of the sequence. The introduced point mutation (S52A) is shown in red.

| **5’ to 3’ sequence** |
| --- |
| cgggctgttttcaaactcataatcctctgggtaagcctctagagtgttgatgtacatatatacacctagatgccctacttaagtggtaatattcaaatagtgttttaaccttgacatgttcctttaatccttaggcctcacgtcactttatagatagtcattattttttaatagtttctctaaaagtgattgcctaagcatcacaaaaatatggagcatataaaaactaaagttgagtcctgggtatccattttctgacttgtaagagatgagactaatatcaatgttactttgttctgttccttcattgtcagggtctacaacattgattatttttagcttaacactttatttttgtttgtttaaatttcagAATGCCGGGGCTAAGTTGTAGATTTTATCAACACAAATTTCCTGAGGTGGAAGATGTAGTGATGGTGAATGTAAGATCCATTGCTGAAATGGGGGCCTATGTCAGCTTGTTGGAgTAcAAcAAtATcGAgGGCATGATTCTTCTTAGTGAATTAgCCcgaCGcCGcATtCGaTCcATtAACAAACTGATCCGAATTGGCAGAAATGAATGTGTTGTTGTCATTAGAGTGGATAAAGAAAAAGgtaagtgaggaaaaaatagttaagaaatataaactataaaactaaagaatttcttattttaaattgtttatttttaaagtatatattataaaatacacctaattaaactactatactttgttagttttctgagtctcaagagacaattaaagaaagaaggtcactcaaggatgagactttactcacagggaaatctagcctctgaactgaatatcaaaaagaccctctaaagcatatttattaattgttcacaaaagttatttttttggcttagtttctcatactaaaagaccctaatatgtttcctgaagggacatccctcctttgcaacttcagtccttattgggtactgtttggactgtttgcagtactcaataattcaagatattcaggtgtgccagaactgactcttgtgaccaaagttacgcaaaagctgtaaagctccttcagacagacaactctgtagctaacagaaaacactgttttctacaatgacttcttcaagatcagagtacttctagaaaactatttcattctactatttacgtagatataatgattctactagaattcttgctacataagttcttttacatacaaaatagttagagaagcacgtgggggctgaggacataaccagtcatttaaatgcttgattacaagctggaagaggtgagttcaatccctgaacccatataaaaaggccaagtgtgatagcataggctggtagtcccgacaactctgggaaaggtggaaatctgggattccagccagccagcctccta |

Mouse *Ddit3* modifications: mCherry repair construct (generated by Gibson assembly). The mCherry coding region is indicated in red and the SV40 PolyA sequence is underlined.

| **5’ to 3’ sequence** |
| --- |
| CCCGGAGAAAGCCTATCAGTTCCACACCCATGCTGCCTGTGTGCCGTACCTGAGTCAGGTTTCCAGCAGCCACAGAAGGTGGCTCACATGGCCTGGACCTCCAGCTCCAGGAGAGCCAATGAATGCTGCTGGCCCCCAGACACTGAATTACATCCGTTTCAGGGTCCTGGCCATGGTGTGCATGTGATCATCTGGACAACTTTTGAGAGTTGGATCTGGCAGGGTCAAAGTCAAGGCTGCTAGGCTTGAGAGGCAGCCATCTCCCCATCCCGACACACCATCATTAGTGTGTGTGCAGGTCAGAGAACAACTTGTGCGAGTTGACTCTTCACCTCCACCCTCTGCCAATGTAGCCTTCAAGGAGTGACAACCCATGCCCTTACCTATCGTGCAAGACCAGTAAATTTTAAATTCTACGTGTTAGAAAAGGGACAAGGTCAGCTCACCGACTGTGGTGAATGGAATGTATGTCCTTTCCAGAACCTGGTCCACGTGCAGTCATGGTGAGCAAGGGCGAGGAGGATAACATGGCCATCATCAAGGAGTTCATGCGCTTCAAGGTGCACATGGAGGGCTCCGTGAACGGCCACGAGTTCGAGATCGAGGGCGAGGGCGAGGGCCGCCCCTACGAGGGCACCCAGACCGCCAAGCTGAAGGTGACCAAGGGTGGCCCCCTGCCCTTCGCCTGGGACATCCTGTCCCCTCAGTTCATGTACGGCTCCAAGGCCTACGTGAAGCACCCCGCCGACATCCCCGACTACTTGAAGCTGTCCTTCCCCGAGGGCTTCAAGTGGGAGCGCGTGATGAACTTCGAGGACGGCGGCGTGGTGACCGTGACCCAGGACTCCTCCCTGCAGGACGGCGAGTTCATCTACAAGGTGAAGCTGCGCGGCACCAACTTCCCCTCCGACGGCCCCGTAATGCAGAAGAAGACCATGGGCTGGGAGGCCTCCTCCGAGCGGATGTACCCCGAGGACGGCGCCCTGAAGGGCGAGATCAAGCAGAGGCTGAAGCTGAAGGACGGCGGCCACTACGACGCTGAGGTCAAGACCACCTACAAGGCCAAGAAGCCCGTGCAGCTGCCCGGCGCCTACAACGTCAACATCAAGTTGGACATCACCTCCCACAACGAGGACTACACCATCGTGGAACAGTACGAACGCGCCGAGGGCCGCCACTCCACCGGCGGCATGGACGAGCTGTACAAGTAATTGTTTATTGCAGCTTATAATGGTTACAAATAAAGCAATAGCATCACAAATTTCACAAATAAAGCATTTTTTTCACTGCATTCTAGTTGTGGTTTGTCCAAACTCATCAATGTATCTTAGTGAGTGAGAATGCTGGTCCTAGGATGGGCGAGCAGAGTGATGGTGTGGGTGCCTATAGCCCCAGTGCTTGTCAAGTATAGGCAAGAGGCTCAGTTCATGGCCAGCCTAAGCTAGAGTTTGAATGTAGCCTATACAAGACCCTGTCTCAAAAACCAAGCAAAAGTAAAACCCCAGGAAACTGGGGGTTTGTATGCCTCTCCTGAACTAATTAATATCTATCTCCCCTTCTTCATTTCCTTAAAGGAAGAATCAAAAACCTTCACTACTCTTGACCCTGCGTCCCTAGCTTGGCTGACAGAGGAGCCAGGGCCAACAGAGGTCACACGCACATCCCAAAGCCCTCGCTCTCCAGATTCCAGTCAGAGTTCTATGGCCCAGGAGGAAGAGGAGGAAGAGCAAGGAAGAACTAGGAAACGGAAACAGAGTGGTCAGTGCCCAGCCCGGCCTGGGAAGCAACGCATGAAGGAGAAGGAGCAGGAGAACGAGCGGAAAGTGGCACAGCTAGCTGAAGAGAACGAGCGG |

Mouse *Ddit3* modifications: NanoLuc-PEST repair construct (generated by complete gene synthesis, IDT). The NanoLuc-PEST coding region is indicated in blue and the SV40 PolyA sequence is underlined.

| **5’ to 3’ sequence** |
| --- |
| GAGCCCGGAGAAAGCCTATCAGTTCCACACCCATGCTGCCTGTGTGCCGTACCTGAGTCAGGTTTCCAGCAGCCACAGAAGGTGGCTCACATGGCCTGGACCTCCAGCTCCAGGAGAGCCAATGAATGCTGCTGGCCCCCAGACACTGAATTACATCCGTTTCAGGGTCCTGGCCATGGTGTGCATGTGATCATCTGGACAACTTTTGAGAGTTGGATCTGGCAGGGTCAAAGTCAAGGCTGCTAGGCTTGAGAGGCAGCCATCTCCCCATCCCGACACACCATCATTAGTGTGTGTGCAGGTCAGAGAACAACTTGTGCGAGTTGACTCTTCACCTCCACCCTCTGCCAATGTAGCCTTCAAGGAGTGACAACCCATGCCCTTACCTATCGTGCAAGACCAGTAAATTTTAAATTCTACGTGTTAGAAAAGGGACAAGGTCAGCTCACCGACTGTGGTGAATGGAATGTATGTCCTTTCCAGAACCTGGTCCACGTGCAGTCATGGTCTTCACACTCGAAGATTTCGTTGGGGACTGGCGACAGACAGCCGGCTACAACCTGGACCAAGTCCTTGAACAGGGAGGTGTGTCCAGTTTGTTTCAGAATCTCGGGGTGTCCGTAACTCCGATCCAAAGGATTGTCCTGAGCGGTGAAAATGGGCTGAAGATCGACATCCATGTCATCATCCCGTATGAAGGTCTGAGCGGCGACCAAATGGGCCAGATCGAAAAAATTTTTAAGGTGGTGTACCCTGTGGATGATCATCACTTTAAGGTGATCCTGCACTATGGCACACTGGTAATCGACGGGGTTACGCCGAACATGATCGACTATTTCGGACGGCCGTATGAAGGCATCGCCGTGTTCGACGGCAAAAAGATCACTGTAACAGGGACCCTGTGGAACGGCAACAAAATTATCGACGAGCGCCTGATCAACCCCGACGGCTCCCTGCTGTTCCGAGTAACCATCAACGGAGTGACCGGCTGGCGGCTGTGCGAACGCATTCTGGCGAATTCTCACGGCTTTCCGCCTGAGGTTGAAGAGCAAGCCGCCGGTACATTGCCTATGTCCTGCGCACAAGAAAGCGGTATGGACCGGCACCCAGCCGCTTGTGCTTCAGCTCGCATCAACGTCTAATTGTTTATTGCAGCTTATAATGGTTACAAATAAAGCAATAGCATCACAAATTTCACAAATAAAGCATTTTTTTCACTGCATTCTAGTTGTGGTTTGTCCAAACTCATCAATGTATCTTAGTGAGTGAGAATGCTGGTCCTAGGATGGGCGAGCAGAGTGATGGTGTGGGTGCCTATAGCCCCAGTGCTTGTCAAGTATAGGCAAGAGGCTCAGTTCATGGCCAGCCTAAGCTAGAGTTTGAATGTAGCCTATACAAGACCCTGTCTCAAAAACCAAGCAAAAGTAAAACCCCAGGAAACTGGGGGTTTGTATGCCTCTCCTGAACTAATTAATATCTATCTCCCCTTCTTCATTTCCTTAAAGGAAGAATCAAAAACCTTCACTACTCTTGACCCTGCGTCCCTAGCTTGGCTGACAGAGGAGCCAGGGCCAACAGAGGTCACACGCACATCCCAAAGCCCTCGCTCTCCAGATTCCAGTCAGAGTTCTATGGCCCAGGAGGAAGAGGAGGAAGAGCAAGGAAGAACTAGGAAACGGAAACAGAGTGGTCAGTGCCCAGCCCGGCCTGGGAAGCAACGCATGAAGGAGAAGGAGCAGGAGAACGAGCGGAAAGTGGCACAGCTAGCTG |

**
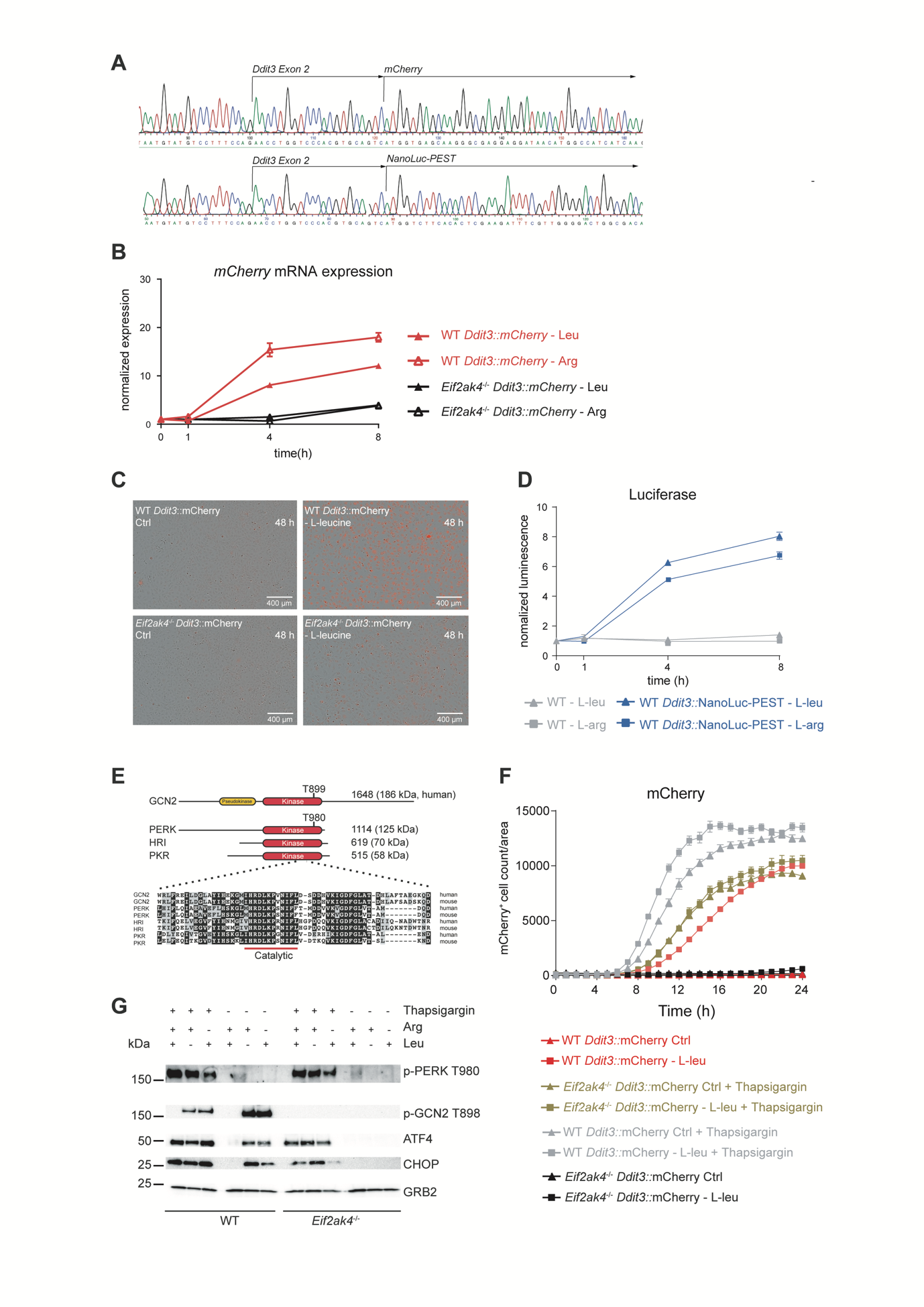
**

**Supplemental Figure 1**

**A** Sanger sequencing analysis of the insertion sites of each reporter (mCherry and NanoLuc-PEST) into the *Ddit3* coding exon (exon 2).

**B** mCherry mRNA expression in leucine or arginine starvation (- Leu, -Arg) across time (1 h, 4 h and 8 h) comparing wild-type *Ddti3*::mCherry 3T3 and GCN2-deficient (*Eif2ak4*^-/-^) cells.

**C** Representative images of the 3T3 mCherry reporter

**D** Luminescence detection of *Ddit3*::NanoLuc-PEST 3T3 cells in leucine or arginine starvation (- Leu, -Arg) across time (1 h, 4 h and 8 h). Parental 3T3 cells (WT, grey) served as internal negative control for assay normalization.

**E** Schematic representation of the aligned sequence similarities of the autophosphorylation sites and the catalytic domains of GCN2, PERK, PKR and HRI (human and mouse). In contrast to HRI, PERK and PKR, GCN2 consists of an additional pseudokinase domain (yellow) next to its kinase domain (red).

**F** mCherry quantification of wild-type *Ddti3*::mCherry and their GCN2-deficient (*Eif2ak4*^-/-^) counterpart cells incubated in media lacking leucine (- L-leu) and treated with 1µM thapsigargin (Tg) for 24 h. mCherry intensity was measured by live-cell imaging (IncuCyte S3) and normalized to cellular confluence. Data are depicted as mean ± SEM of three technical replicates and represent one of three independent experiments.

**G** GCN2 versus PERK ISR activation specificity. 3T3 wild-type (WT) or GCN2-deficient (*Eif2ak4*^-/-^) cells were cultured in media lacking Leu or Arg for 8 h in the absence or presence of 1 μM thapsigargin (Tg) to induce ER stress and PERK activation measured by immunoblotting for the activated forms of GCN2 (p-GCN2 T989) or PERK (p-PERK T980) along with ATF4 and CHOP as markers of ISR activation. GBR2 served as the loading control.

**
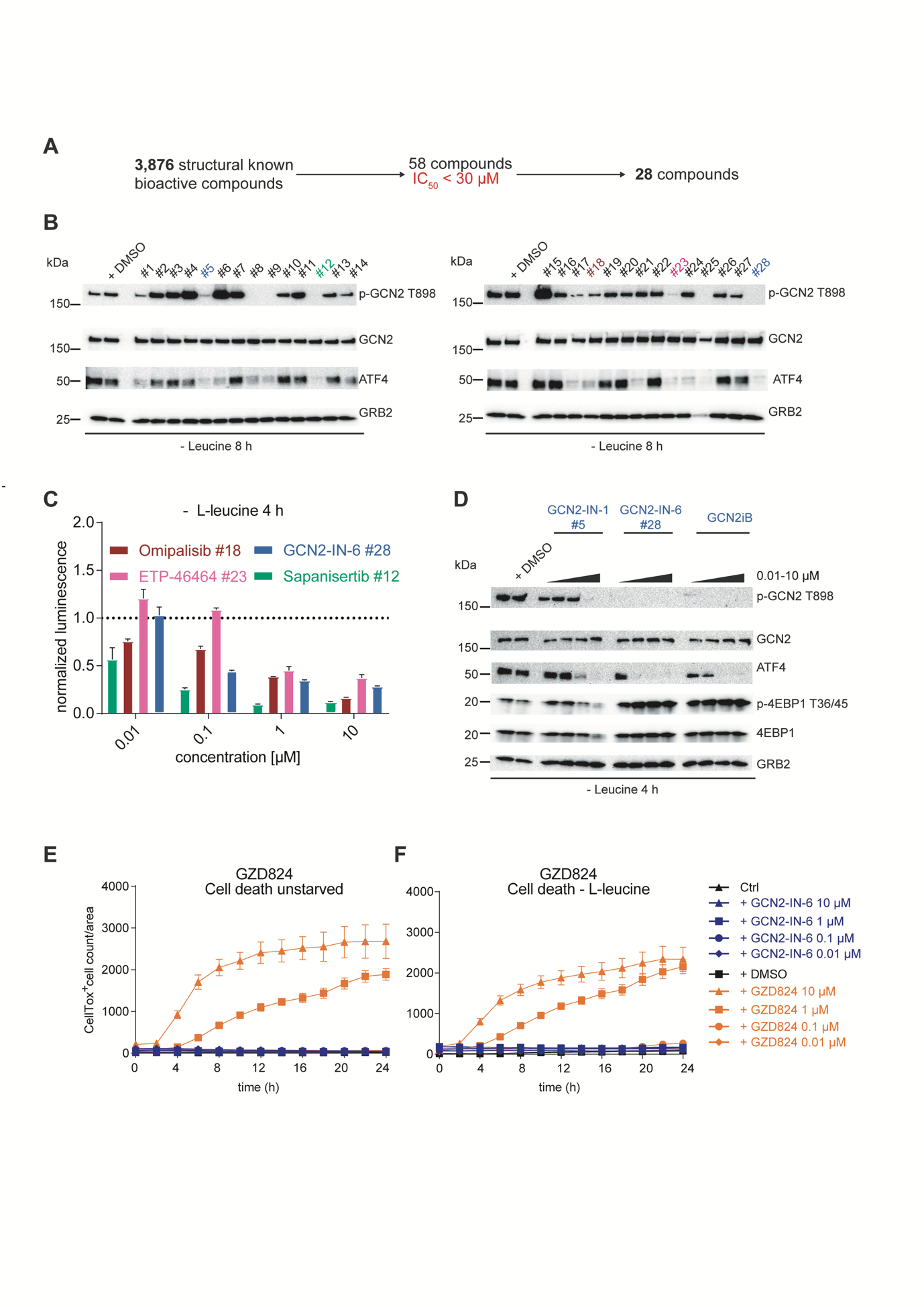
**

**Supplemental Figure 2**

**A** Overview of the primary GCN2 inhibitor screen.

**B** GCN2 pathway analysis of 28 compounds (Table S1). 3T3 cells were incubated in media lacking Leu for 8 h without or with DMSO (dimethyl sulfoxide) as control in comparison to a fixed amount of compound (10 μM) added at the initiation of L-leu starvation.

**C** Dose-dependent inhibition using *Ddti3*::NanoLuc-PEST 3T3 cells normalized to the maximal signal in L-leu starved *Ddti3*::NanoLuc-PEST 3T3 cells (dotted line).

**D** GCN2 pathway immunoblotting analysis for three GCN2i. Data are representative of three independent experiments.

**E**, **F** Dose- dependent quantification of cellular toxicity of GZD824 (compound #25) treatment in comparison to GCN2-IN-6 in unstarved and leucine starved conditions. Cell death was measured by live-cell imaging in the IncuCyte S3 using CellTox green staining and normalized to cellular confluence. Data are depicted as mean ± SEM of three technical replicates and represent one of three independent experiments.


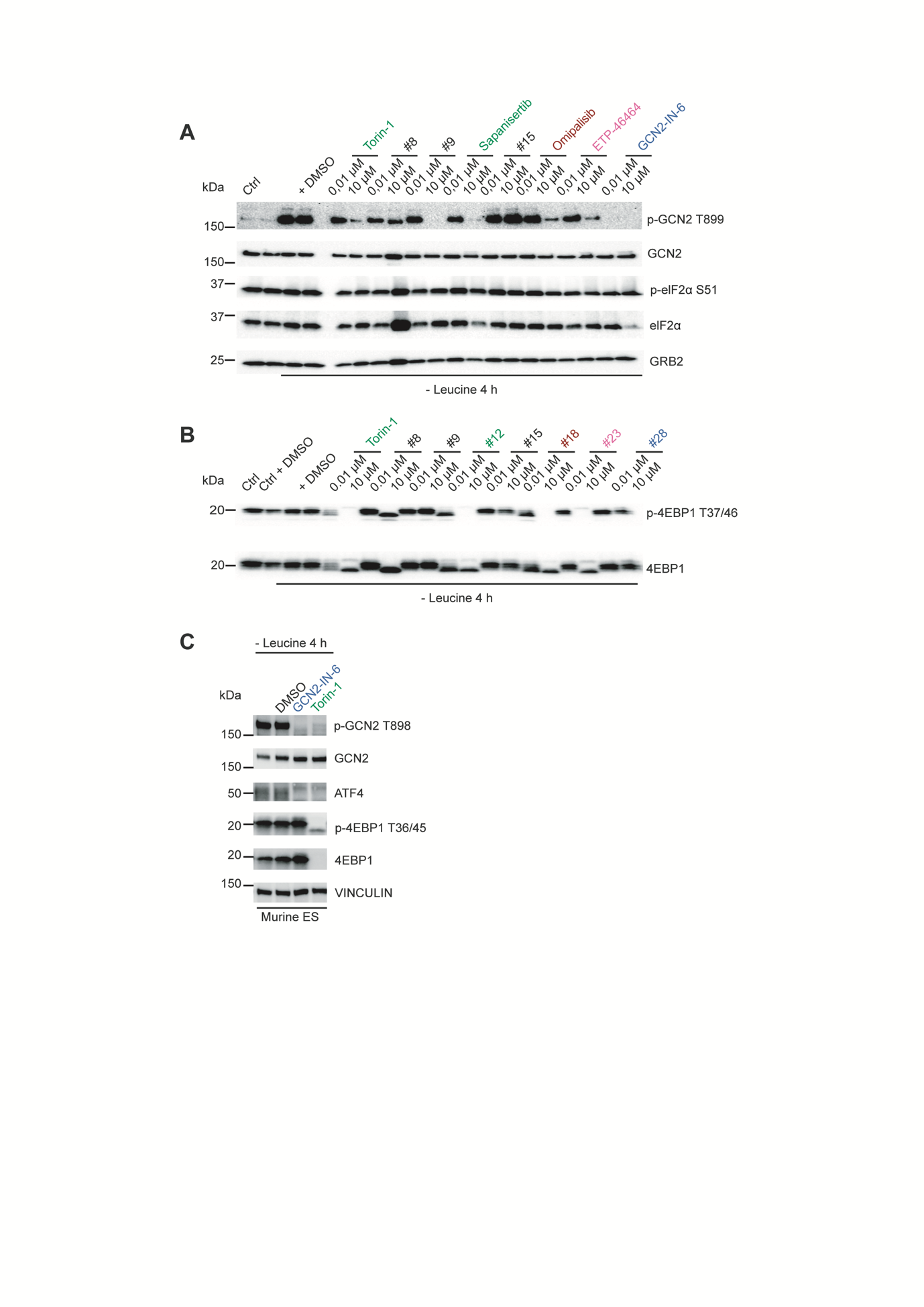


**Supplemental Figure 3**

**A**, **B** GCN2 pathway analysis of selected compounds (Table S1) on GCN2 (A) or mTORC1 (B) in HeLa cells incubated in media lacking Leu for 4 h without or with DMSO (dimethyl sulfoxide) as control in comparison to a fixed amount of compound (0.01 or 10 μM) added at the initiation of L-leu starvation.

**C** GCN2 and mTORC1 pathway analysis in murine ES cells starved of leucine for 4 h.


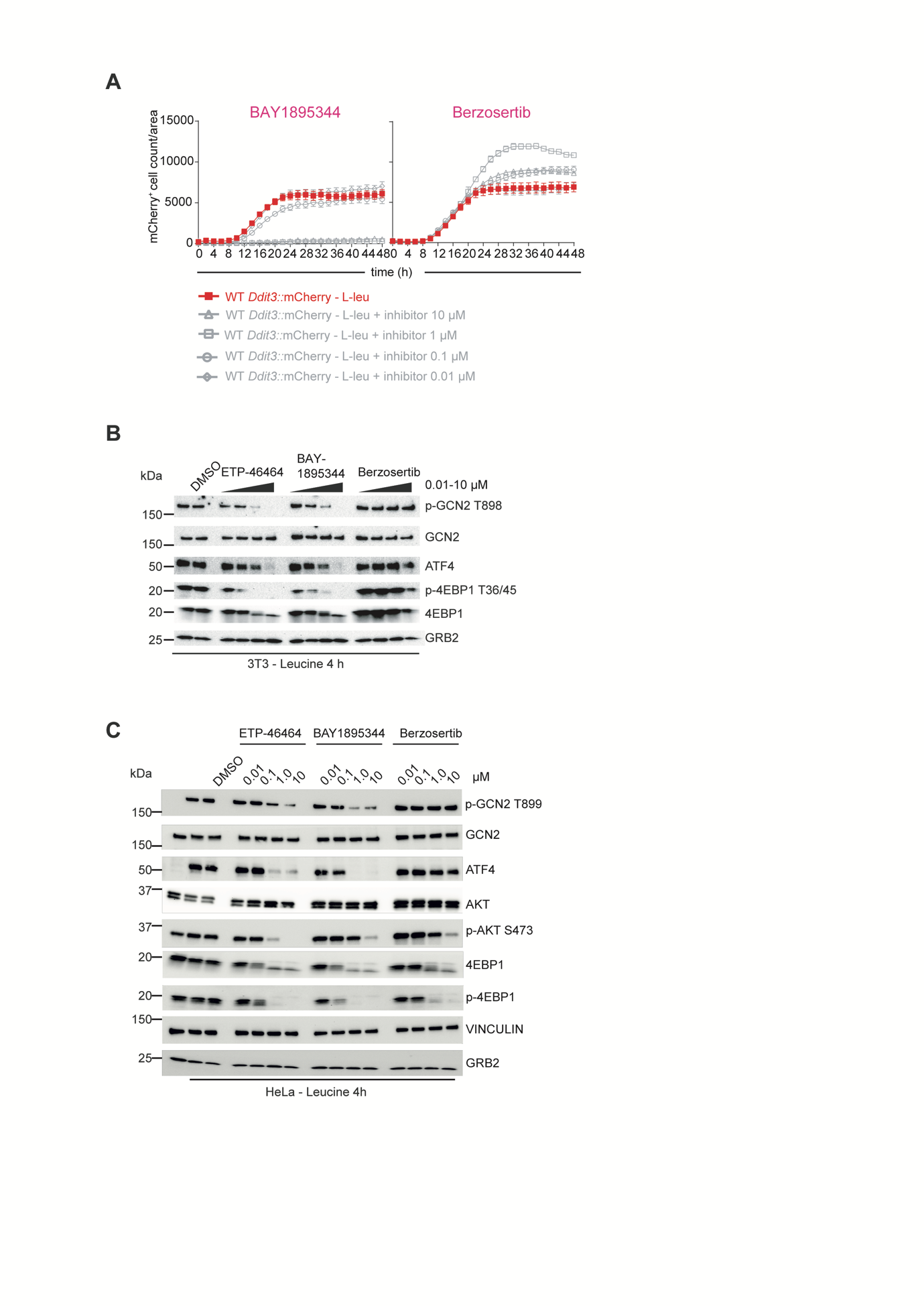


**Supplemental Figure 4**

**A** Kinetics of ATRi. Different doses of ATRi BAY1895344 or berzosertib were tested in *Ddti3*::mCherry clonal 3T3 cells starved with Leu-free media for 48 h and mCherry expression measured in the live-cell imager IncuCyte S3 and normalized to cellular confluence. Data are depicted as mean ± SEM of three technical replicates and represent one of three independent experiments.

**B, C** GCN2 pathway immunoblotting analysis of ATRi (ETP-46464, BAY1895344 or berzosertib). - Leu 4 h and - Leu 4 h + DMSO are controls of 3T3 or HeLa cells treated for 4 h with Leu-free media to induce the GCN2-ISR as the baseline control. Data are depicted as one of three independent experiments.

**
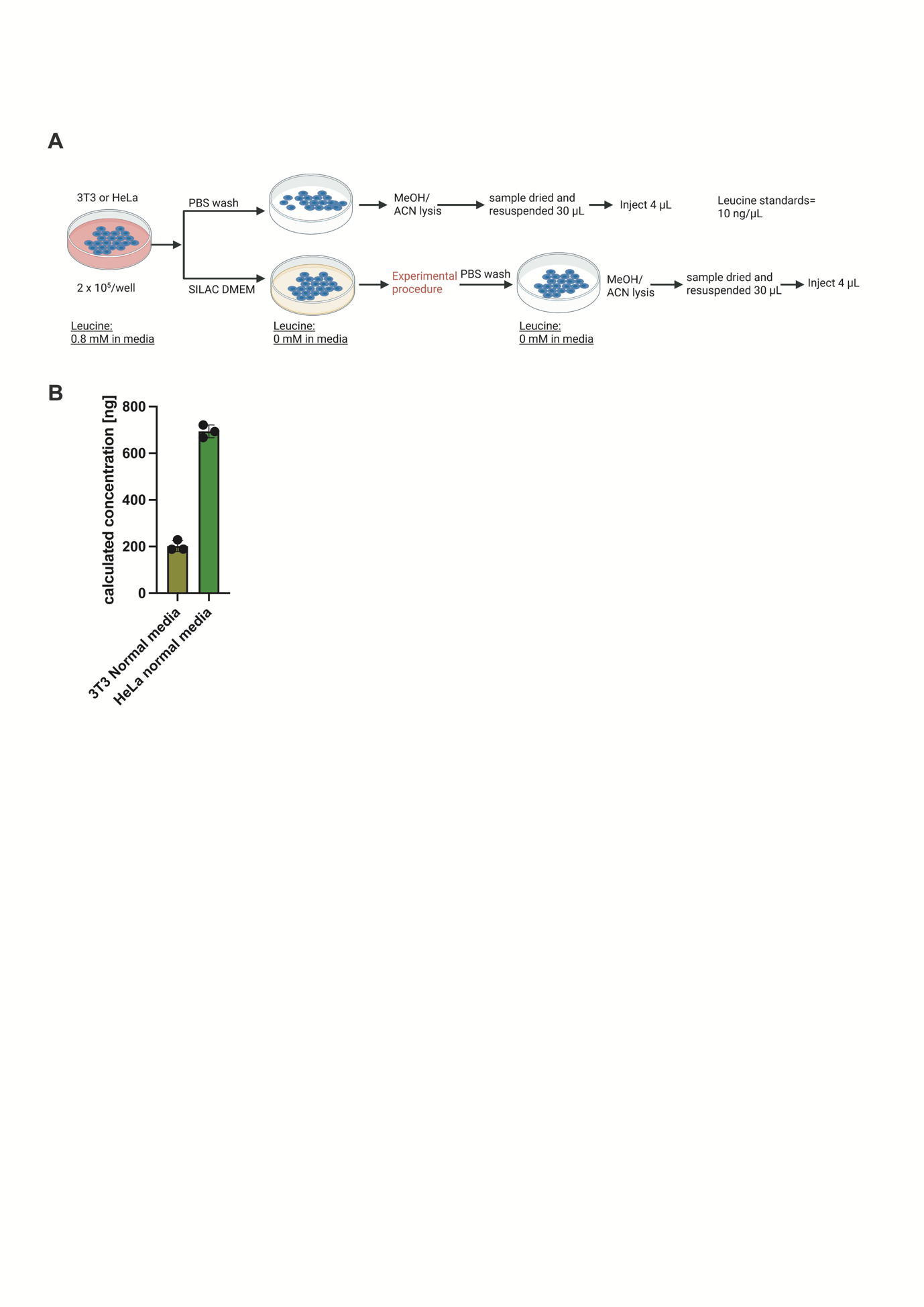
**

**Supplemental Figure 5**

**A** Workflow for the MS-based detection of leucine.

**B** Baseline reading of leucine inside 3T3 or HeLa cells following washing at lysis where cells were cultured in complete media.

**
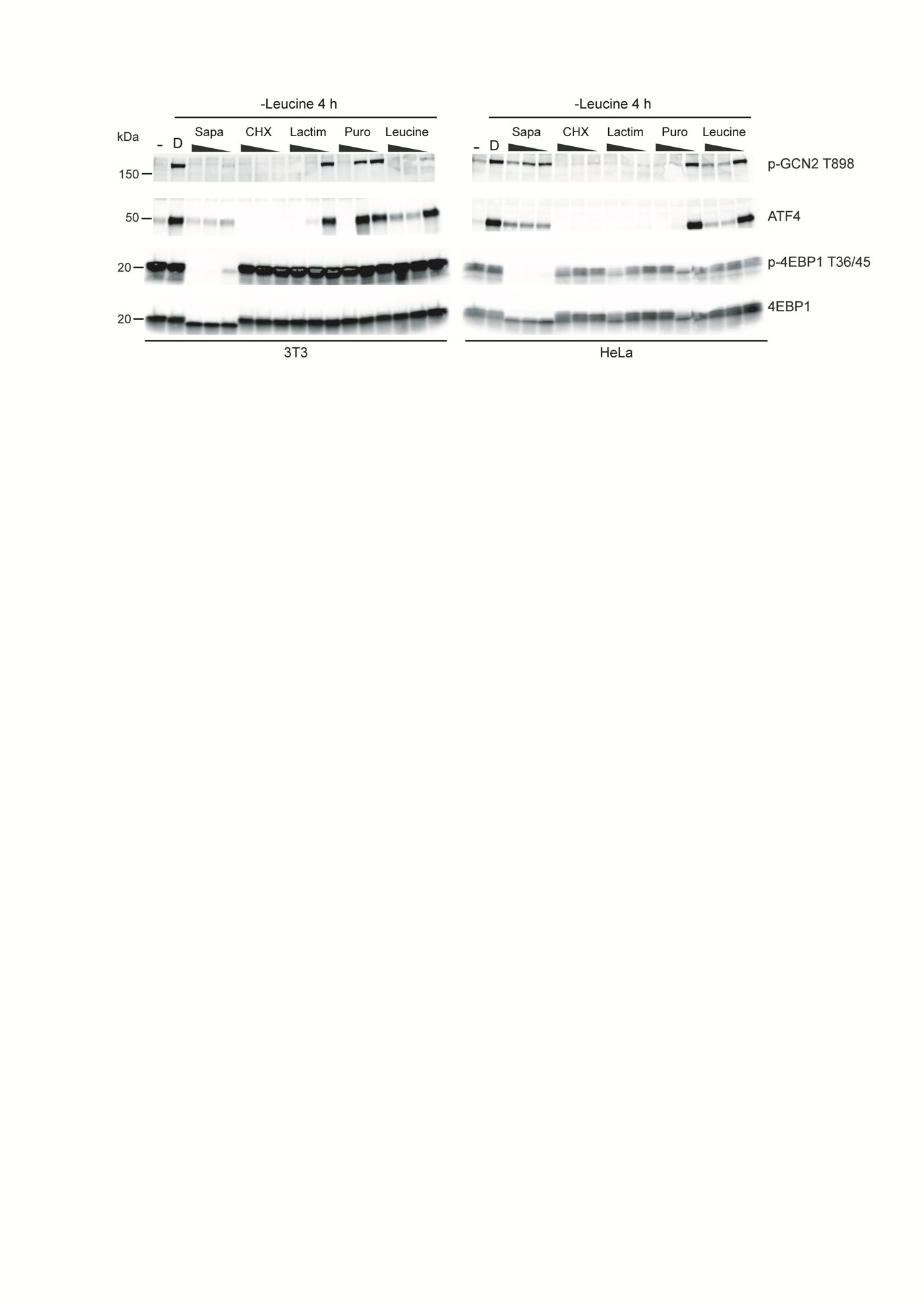
**

**Supplemental Figure 6**

Translation inhibitors reverse GCN2 activation. 3T3 or Hela cells were cultured in normal media (single left lane) or media lacking Leu for 4 h to induce GCN2 activation. At time zero, sapanisertib, cycloheximide (CHX), lactimidomycin (Lactim), puromycin (Puro) or leucine was added in the concentrations detailed in the Methods for 4 h. Lysates were probed for activation of GCN2 (p-GCN2 T898), ATF4 as a marker of the ISR or p-4EBP1 T36/45 as an indicator of mTORC1 activity. GRB2 was used as a loading control. Note that translation inhibitors do not affect mTORC1 activity and have a differential effect of ATF4 (which is newly transcribed and translated after GCN2 activation). Data are representative of two independent experiments for HeLa cells and five experiments for 3T3 cells.
